# Superconducting Phases in Lithium Decorated Graphene LiC_6_

**DOI:** 10.1038/s41598-018-32050-9

**Published:** 2018-09-14

**Authors:** Rouhollah Gholami, Rostam Moradian, Sina Moradian, Warren E. Pickett

**Affiliations:** 1 Physics Department, Faculty of Science Razi University, Kermanshah, Iran; 2 Nano science and nano technology research center, Razi University, Kermanshah, Iran; 3 Department of Electrical and Computer Engineering, University of Central Florida, Orlando, Florida USA; 4 Department of Physics UC Davis, One Shield Avenue, Davis, CA 95616 USA

## Abstract

A study of possible superconducting phases of graphene has been constructed in detail. A realistic tight binding model, fit to ab initio calculations, accounts for the Li-decoration of graphene with broken lattice symmetry, and includes *s* and *d* symmetry Bloch character that influences the gap symmetries that can arise. The resulting seven hybridized Li-C orbitals that support nine possible bond pairing amplitudes. The gap equation is solved for all possible gap symmetries. One band is weakly dispersive near the Fermi energy along Γ → *M* where its Bloch wave function has linear combination of ${d}_{{x}^{2}-{y}^{2}}$ and *d*_*xy*_ character, and is responsible for ${d}_{{x}^{2}-{y}^{2}}$ and *d*_*xy*_ pairing with lowest pairing energy in our model. These symmetries almost preserve properties from a two band model of pristine graphene. Another part of this band, along *K* → Γ, is nearly degenerate with upper *s* band that favors extended *s* wave pairing which is not found in two band model. Upon electron doping to a critical chemical potential *μ*_1_ = 0.22 *eV* the pairing potential decreases, then increases until a second critical value *μ*_2_ = 1.3 eV at which a phase transition to a distorted *s*-wave occurs. The distortion of *d*- or s-wave phases are a consequence of decoration which is not appear in two band pristine model. In the pristine graphene these phases convert to usual *d*-wave or extended *s*-wave pairing.

## Introduction

Two dimensional superconducting phases have become of great interest since the discovery of the high temperature superconducting (HTS) cuprates and subsequent finding of Fe-pnictide and -chalcogenide HTSs. Interest was re-invigorated by the discovery of superconductivity onsets up to 75 K in single layer FeSe grown on *SrTiO*_3_ and related substrates^1,2^. With the enormous research activity focused on graphene in recent years, it is not surprising that graphene-based superconductivity has become an active area of research. Very recently superconductivity up to 1.7 K has been reported^3^ in magic angle bilayer graphene, which will buttress activity on two dimension superconductors and especially the related type that we discuss here.

Superconductivity has been known for some time in intercalated graphite compounds such as *C*_6_*Ca* and *C*_6_*Yb* ^4^. With the many remarkable properties of graphene, it has been anticipated that doping by gating or by decorating with electro-positive elements, thereby moving the chemical potential away from the Dirac points, might induce superconductivity. However, graphene decorated with alkali metals has three valence bands with one weakly dispersive band near Fermi energy. Due to this flat band, there are additional available states around the Fermi level and the required pairing potential is reduced.

Discussion of superconductivity in doped graphene has been primarily within theoretical models, as we review below, but some encouraging data have been reported. Experimental evidence for a superconducting gap in Li-decorated monolayer graphene around 6 K has been reported by Ludbrook *et al*. based on angle-resolved photoemission spectroscopy^5^ (ARPES). Scanning tunneling spectroscopy (STS) was applied by Palinkas *et al*.^6^ to graphene suspended on tin nanoparticles, who concluded that superconductivity is induced in the graphene layer. Evidence of superconductivity in Li-decorated few layer graphene at 7.4 K has been reported by Tiwari and collaborators^7^. Low temperature mobility of K and Li atoms on graphene was observed by Woo *et al*., and suggest that mobility may persist at lower temperatures^8^, which would provide new challenges for theory.

Various mechanisms of pairing have been proposed. Uchoa and Castro-Neto modeled pristine and doped graphene with electron-phonon coupling or plasmon mediated in mind^9^. Repulsive electron-electron interactions were modeled by Nandkishore and collaborators^10,11^. Beginning from pristine graphene, varying the chemical potential leads to dominant chiral singlet ${d}_{{x}^{2}-{y}^{2}}+i{d}_{xy}$ pairing for nearest neighbor interaction, according to Black-Schaffer *et al*.^12^ a triplet *f*-wave state has been proposed to arise from next-nearest neighbor interaction with chemical potential near van Hove peak^13^. Both chiral and conventional *p*-wave states in graphene have been discussed^14^, with the many pictures raising various possibilities but little of a certain nature.

More specific predictions have begun to appear. Profeta *et al*. predicted^15^ based on Eliashberg theory that decoration by electron donating atoms such as Ca and Li would make single layer graphene superconducting, with modest critical temperatures in the 1–8 K range. In somewhat related work, Wong *et al*. have predicted^16^ from an ab initio treatment a critical temperature around T_*c*_ = 14 K for carbon nanotubes, which was increased to above 100 K for a certain type of carbon ring.

Expectations of adjusting the chemical potential include gating, but the main focus has been on decoration of graphene by electropositive atoms, viz. alkalis or alkaline earths. Charge migration from such decorating atoms to the graphene layer will affect the C-C bonding, leading to contraction or expansion of the graphene hexagons that are centered by the decorating atoms, thus breaking the symmetry of C-C hopping integrals around the honeycomb loop. This asymmetric graphene layer will be referred to in this paper as “shrunken graphene”. Taking LiC_6_ for illustration, each cell site has six C atoms in a hexagon with an alkaline atom lying above the center of the hexagon. The C *π* orbitals and alkaline atom’s *s*-orbital hybridize to give seven “molecular” orbitals. For two dimensional graphene-like structures effects, differences in nearest neighbour hopping integrals affect the band structure near the important Dirac point, which is folded back to the Γ point of the shrunken graphene superlattice investigated by Hou *et al*.^17^ and Long-Hua *et al*.^18^. For such systems not even the full analytic tight-binding band structure has yet been reported. The intent here is to extend study of this system, with representative LiC_6_, from the underlying electronic structure to investigation of the possible superconducting phases.

The organization of the paper is as follows. In Sec. II the interacting seven orbital model Hamiltonian is presented. The exact band structure of the normal state of this shrunken graphene system is described in Sec. III. Perturbation theory is applied to obtain the band structures in analytic form. Applying the Hubbard model and minimizing free energy of the superconductor state, we obtain in Sec. IV the gap equations and approximate critical temperature. These equations are solved analytically to establish the possible pairing symmetries and other properties of the superconducting states. A summary is provided in Sec. V.

## Model Hamiltonian

**Figure 1:**
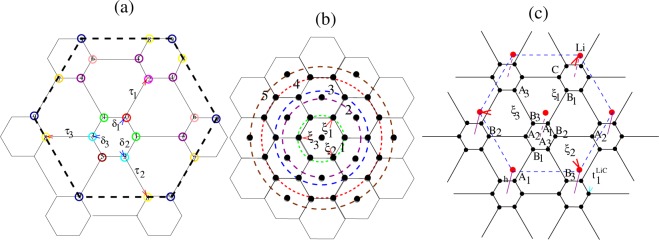
(**a**) Schematic diagram of the shrunk graphene lattice, with the distortion emphasized. (**b**) The hexagonal Li sheet, indicating the circles that Li neighbors lie on. (**c**) Diagram of the graphene decorated by lithium. The red Li atoms lie above the centers of the C hexagons.

Because the unit cell contains several atoms with important specific aspects, we provide many of the details of the expressions that can be obtained analytically. LiC_6_, as illustrated in Fig. 1, consists of a graphene layer decorated by a lithium layer in which Li atoms are located at the center of a carbon hexagon surrounded by six empty center hexagons. The height of Li above the carbon layer is calculated to be *h*_*z*_ = 1.85, somewhat smaller than the value 1.93 Å obtained by Profeta *et al*.^15^. The nearest Li-C distances are *h* = 2.40. Since the Li 2*s* orbital energy is higher than the C 2*p*_*z*_ orbital, charge transfer occurs. It is calculated that 0.685*e* from Li transfers to the six C atoms equally^19^. The positive Li ion and negative C ion provide a relative Coulomb (Madelung) shift in site potentials of the two atoms.

The attractive interaction between Li and C ions after charge transfer contracts the Li-C distance and reduces the C-C bond lengths in the Li-centered hexagon to *a*_1_ = 1.425, while the bond length of nearest neighbor C atoms in different hexagons is slightly larger at *a*_2_ = 1.426. For Ca instead of Li, this difference should be larger, hence we keep these lengths distinct. The hopping integral between short-bond carbons is *t*_1_, with that between stretched carbon sites is denoted ${t}_{1}^{^{\prime} }$. We refer to this broken symmetry situation as “shrunk graphene”. The difference in hopping amplitudes indicates that the new Li-C hopping parameter is the central new feature in LiC_6_ compared to graphene. Symmetry breakdown leads to the opening of a small energy gap at the Γ point.

The lattice then becomes a two dimensional hexagonal Bravais lattice with seven atomic sites. These will be labeled as *A*_1_, *A*_2_, *A*_3_, *B*_1_, *B*_2_, *B*_3_ and *Li*, as illustrated in Fig. 1. The Hamiltonian of this system is

1 $$\hat{H}=-\,\sum _{i\alpha }\sum _{j\beta ,\sigma }{t}_{i\alpha ,j\beta }^{\sigma ,\sigma }{\hat{c}}_{i\alpha \sigma }^{\dagger }{\hat{c}}_{j\beta \sigma }+\sum _{i\alpha ,\sigma }\,({\varepsilon }_{i\alpha }-{\mu }_{o}){\hat{n}}_{i\alpha \sigma }+\frac{1}{2}\,\sum _{i\alpha ,\sigma }\,\sum _{j\beta ,\sigma ^{\prime} }\,{U}_{i\alpha ,j\beta }^{\sigma ,\sigma \text{'}}{\hat{n}}_{i\alpha \sigma }{\hat{n}}_{j\beta \sigma ^{\prime} }={\hat{H}}_{N}+{\hat{H}}_{P}.$$

Here *H*_*N*_ and *H*_*P*_ denote the non-interacting and interaction Hamiltonians respectively. In these expressions *α* and *β* run over *A*_*i*_, *B*_*i*_ and *Li*. Here ${\hat{c}}_{i\alpha \sigma }^{\dagger }$, ${\hat{c}}_{i\alpha \sigma }$ are creation and annihilation operators of an electron with spin *σ* on subsite *α* of *i*th lattice site, and ${\hat{n}}_{i\alpha \sigma }={\hat{c}}_{i\alpha \sigma }^{\dagger }{\hat{c}}_{i\alpha \sigma }$ is the electron number operator. The noninteracting chemical potential is *μ*_0_ and *t*_*iα*,*jβ*_ is the hopping integral from the *α* site of *i*th cell to the *β* site of *j*th cell. We denote the on-site energy by *ε*_*α*_.

The interaction stated above corresponds to an extended (negative *U*) Hubbard model, which allows a variety of phenomenological values to be chosen and studied. It is largely for this reason that we provide substantial detail of the underlying, non-interacting C-Li lattice and electronic structure. The interactions that we study are introduced in Sec. IV.

## Normal State of LiC_6_

Many studies of graphene rely on tight binding parametrization of the band structure. The early parametrization of Wallace^20^ already employed both first and second neighbors. Extensions in various ways have followed^21,22^, culminating in the application of Wannier functions by Jung and MacDonald^23^ to provide simple but realistic five parameter model and a more accurate but more involved 15 parameter model. Our aim in this section is to construct a realistic seven band model for distorted LiC_6_, while also developing the formalism to allow exploration of superconducting phases once the interaction has been included.

The distortion of the graphene layer to shrunken graphene and the coupling to Li requires a considerable generalization of the underlying tight binding model Hamiltonian, and many of the details are relegated to appendices. The Hamiltonian of non-interacting LiC_6_ is

2 $${\hat{H}}_{N}=-\,\sum _{i\alpha }\,\sum _{j\beta ,\sigma }\,{t}_{i\alpha ,j\beta }^{\sigma ,\sigma }\,{c}_{i\alpha \sigma }^{\dagger }\,{c}_{j\beta \sigma }+\sum _{i\alpha ,\sigma }\,({\varepsilon }_{i\alpha }-{\mu }_{o}){\hat{n}}_{i\alpha \sigma }.$$

Eq. 2 incorporates broken symmetries in the on-site energies, hopping integrals, and bond lengths. Here, it has been assumed that on site energies ${\varepsilon }_{{A}_{i}}={\varepsilon }_{A}$ and ${\varepsilon }_{{B}_{i}}={\varepsilon }_{B}$. It is diagonalized in terms of Bloch eigenfunction of the form Eq. A.2. In matrix representation, the equation for the coefficients becomes3where ${d}_{ci}(\overrightarrow{k})$, ${\varepsilon }_{i}(\overrightarrow{k})$, $\beta (\overrightarrow{k})$, $\theta (\overrightarrow{k})$, $\gamma (\overrightarrow{k})$, ${d}_{i}(\overrightarrow{k})$ and ${\tau }_{i}(\overrightarrow{k})$ functions are defined in Supplementary Materials Eqs A.7, A.8, A.9 and A.10 respectively. For general $\overrightarrow{k}$ vectors, it is challenging to obtain an exact analytical expression for the full Hamiltonian in Eq. 3 and it would not be transparent anyway. However, analytical expression for Eq. 3 can be achieved in two steps. Since hopping from Li atoms to nearest neighbor carbon sites ${t}_{1}^{LiC}$ is small with respect to C-C nearest neighbor hopping *t*_1_, by first neglecting the lithium-carbon hopping ${t}_{1}^{LiC}\to 0$, first column and row of the Hamiltonian matrix in Eq. 3 are omitted, the remaining part given by Eq. B.1 is uncoupled shrunken graphene Hamiltonian which can be diagonalized exactly to obtain *E*_*sh*,*n*_. Finally, Li-C coupling is taken into account by perturbation theory to obtain eigenvalues *E*_*n*_, as presented in the appendices.

### Uncoupled *C*_6_ Dispersion Relations

**Figure 2:**
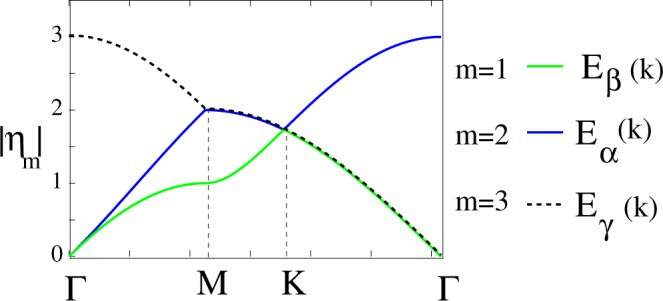
A plot of the dispersion expressions $$|{\eta }_{m}(\overrightarrow{k})|$$, the three folded branches of pristine π^*^ band structure in the mini-Brillouin zone of graphene C_6_. The Bloch wave character of *E*_*β*_ is $${f}_{d}^{1}|d-ip\rangle +{f}_{p}^{1}|p-id\rangle $$, of the *E*_*α*_ is $${f}_{d}^{2}|d+ip\rangle +{f}_{p}^{2}|p+id\rangle $$ and for *E*_*γ*_ is *f*_*s*_|*s*〉 + *f*_*f*_|* f*〉. Here we use abbreviated notation $${f}_{d}^{1(2)}|d\pm ip\rangle =$$$${f}_{{d}_{{x}^{2}-{y}^{2}}}^{1(2)}(|{d}_{{x}^{2}-{y}^{2}}\rangle \pm i|{p}_{x}\rangle )$$ and $${f}_{p}^{1(2)}|p\pm id\rangle ={f}_{{p}_{y}}^{1(2)}(|{p}_{y}\rangle \pm i|{d}_{xy}\rangle )$$.

By first neglecting the lithium-carbon hopping, ${t}_{1}^{LiC}\to 0$, the uncoupled shrunken graphene Hamiltonian given by Eq. B.1 can be diagonalized exactly. Even though Li-C hopping has been neglected but still remaining part of shrunken Hamiltonian in the most general case, include broken symmetries in the hopping integrals, bond lengths and on-site energies. The non trivial eigenvalues of uncoupled shrunken graphene Hamiltonian in general form are given by

4 $${E}_{sh,ml}\,({t}_{i},{\overrightarrow{\xi }}_{i},\overrightarrow{k})=-\,\,{\mu }_{o}+\alpha (\overrightarrow{k})+{u}_{m}{{\rm{\Pi }}}_{0}(\overrightarrow{k})+{u}_{m}^{\ast }{{\rm{\Pi }}}_{0}^{\ast }(\overrightarrow{k})+\frac{1}{2}[{\varepsilon }_{A}+{\varepsilon }_{B}+{(-\mathrm{1)}}^{l}\sqrt{{({\varepsilon }_{A}-{\varepsilon }_{B})}^{2}+4{w}_{m}(\overrightarrow{k})}]$$

with details presented in Supplementary Materials Appendix B. However, the obtained equations are often complicated. To provide insight into the method, uncoupled shrunken graphene Hamiltonian can diagonalized in some particular cases. The Brillouin zone (BZ) of C_6_ is one third of that of graphene, with the Dirac points folded back to the Γ point. In this mini-BZ, the two *π* bands of pristine graphene i.e. *E*_±_ = ±*t*_1_|*η*_0_| folds to six branches as illustrated in Fig. 2. These branches are solutions of Eq. B.1 in the limited case of pristine which in the nearest neighbor approximation they are given by,

5 $$\begin{array}{rcl}{E}_{\gamma }^{\pm }(\overrightarrow{k}) & = & \pm {t}_{1}|{\eta }_{0}(\overrightarrow{k})|,\,{\eta }_{0}(\overrightarrow{k})={e}^{i\overrightarrow{k}.{\overrightarrow{\delta }}_{1}}+{e}^{i\overrightarrow{k}.{\overrightarrow{\delta }}_{2}}+{e}^{i\overrightarrow{k}.{\overrightarrow{\delta }}_{3}}\\ {E}_{\beta }^{\pm }(\overrightarrow{k}) & = & \pm {t}_{1}|{\eta }_{1}(\overrightarrow{k})|,\,{\eta }_{1}(\overrightarrow{k})={e}^{i\overrightarrow{k}.{\overrightarrow{\delta }}_{1}}+{e}^{i\frac{4\pi }{3}}{e}^{i\overrightarrow{k}.{\overrightarrow{\delta }}_{2}}+{e}^{i\frac{2\pi }{3}}{e}^{i\overrightarrow{k}.{\overrightarrow{\delta }}_{3}}\\ {E}_{\alpha }^{\pm }(\overrightarrow{k}) & = & \pm {t}_{1}|{\eta }_{2}(\overrightarrow{k})|,\,{\eta }_{2}(\overrightarrow{k})={e}^{i\overrightarrow{k}.{\overrightarrow{\delta }}_{1}}+{e}^{i\frac{2\pi }{3}}{e}^{i\overrightarrow{k}.{\overrightarrow{\delta }}_{2}}+{e}^{i\frac{4\pi }{3}}{e}^{i\overrightarrow{k}.{\overrightarrow{\delta }}_{3}}.\end{array}$$

Exact analytical solutions for pristine graphene wherein next neighbor hopping integrals are taken into account are presented in Supplementary Materials Eqs B.7 and B.8. As shown in Fig. 2 one sees that ${E}_{\beta }^{\pm }(\overrightarrow{k})$ is weakly dispersive near the van Hove singularity at the saddle points *M* at 3/8 or 5/8 filling (0.25 electron per carbon doping), this band plays a major role in the formation of superconductivity in graphene. Also, one can observe that the band structure is four-fold degenerate at the charge neutral Dirac points. Solution of the Schrödinger equation for pristine graphene in the mini-BZ has another advantage: the Bloch-wave symmetry character of each branch can be distinguished. The Bloch coefficients of the branch labeled by *E*_*γ*_ are of *s*-wave character, ${C}_{{A}_{i}}=(1,\,1,\,1)$ while for those labeled as *E*_*α*_ and *E*_*β*_ are of the form *d* ± *id* -wave i.e. ${C}_{{A}_{i}}=(1,\,{e}^{\pm i\frac{i2\pi }{3}},\,{e}^{\pm i\frac{i4\pi }{3}})$ as illustrated in Fig. 2 and demonstrated in more detail in Appendix B, Eqs B.4 and B.6. This becomes important when it is shown that different superconducting phases of graphene in a variety of doping regimes are due to electron pairing in each of these branches.

Decoration of graphene with metals reduces symmetries that lead to removal of bands degeneracy in some regions. While decoration causes expansion and contraction of bonds length in three inequivalent directions in the honeycomb lattice i.e. $|{\overrightarrow{\tau }}_{i}|\ne |{\overrightarrow{\delta }}_{i}|$, eigenenergies ${E}_{sh,ml}({t}_{i},{\overrightarrow{\xi }}_{i},\overrightarrow{k})$ in Eq. 4 do not depend on the bond lengths ${\overrightarrow{\tau }}_{i}$ and ${\overrightarrow{\delta }}_{i}$ separately but are functions of *LiC*_6_ lattice bases length $|{\overrightarrow{\xi }}_{i}={\overrightarrow{\tau }}_{i}+2{\overrightarrow{\delta }}_{i}|$, so symmetry breakdown of bond lengths does not break symmetries of bands. Symmetry reduction of hopping integrals removes degeneracies occurring in pristine graphene band structure, with the most important effect being to open a gap ${E}_{g}=2|{t}_{1}^{\prime} -{t}_{1}|$ at the Dirac point which has been folded back to the Γ point. This gap arises from symmetry breaking of the nearest neighbor hopping and dose not affected by the other next neighbors hopping nor by the Li-C hopping integral. Comparison with DFT band structures gives *E*_*g*_ = 0.36 *eV*. Another gap can arise at the Γ point because of symmetry breaking of on-site energies *ε*_*A*_ ≠ *ε*_*B*_, seen from Eq. 4. For the case ${t}_{1}={t}_{1}^{\prime}$ the gap becomes 2|*ε*_*A*_ − *ε*_*B*_|. In Li decorated graphene that we consider here, all carbon on-site energies are equal so this type gap does not arise.

While for folded but pristine graphene Bloch wave solutions are pure *s*-wave or chiral *d* ± *id*-wave and there are no mixed states, when symmetries in hopping integrals are broken by decoration, Bloch functions are linear combinations of all these phases, Eq. B.6. Equation B.7 demonstrates that for a general $\overrightarrow{k}$ all probabilities are equal in pristine graphene i.e. $|{C}_{{A}_{i}}({E}_{m}){|}^{2}=|{C}_{{B}_{i}}({E}_{m}){|}^{2}=\frac{1}{6}$. In shrunken graphene these probabilities are $\overrightarrow{k}$ dependent and unequal in general. It will be seen that these small deviations influence the superconducting gap equation symmetries.

### Coupled LiC_6_ Dispersion Relations

Li-C hopping adds a perturbation term to the shrunken graphene Hamiltonian. Obtaining exact dispersions from Eq. 3 is very challenging, so perturbation theory is applied to obtain approximate solutions, as presented in Appendix C. However, to get some insight into effects of the coupling, Eq. 3 can be solved exactly at the Γ point. At $\overrightarrow{k}$ = 0 only the isolated Li band, *E*_*Li*,0_(0) and the lowest valance band, *E*_*sh*,6_(0), are mutually affected. The energies of these bands are, with *E*_0_(0) ≡ *E*_+_, *E*_6_(0) ≡ *E*_−_,

6 $${E}_{\pm }(0)=\frac{1}{2}({E}_{Li,0}(0)+{E}_{sh,6}(0))\pm \sqrt{\frac{1}{4}{[{E}_{Li,0}(0)-{E}_{sh,6}(0)]}^{2}+6{({t}_{1}^{LiC})}^{2}}$$

and other shrunk graphene bands given by (Supplementary) Eq. B.5 remain unchanged. Comparing the fit results from DFT to these equations suggests that ${t}_{1}^{Li-C}$ is in the 0.3–0.5 eV range, and other next neighbor hopping from Li atoms to C sites are negligible.

There are two critical points in the pure graphene band structure which are affected by decoration and become important: the charge neutrality Dirac points folded at the Γ point, and the van Hove singularity at the *M* point. We define a hopping integral symmetry breaking index, ${w}_{t}=\frac{{t}_{1}^{\prime} }{{t}_{1}}\ne 1$ indicates the degree of symmetry breaking. The difference in Li and C on-site energies can be considered to reflect the amount of doping. The Dirac points affected by *w*_*t*_ open a small gap *E*_*g*_ at Γ, which does not depend on ${t}_{1}^{LiC}$. Depending on doping level, Li-C hopping affects the band structure near the points that the isolated Li band ${E}_{Li\mathrm{,0}}(\overrightarrow{k})$ and uncoupled shrunken graphene bands intersect. These impurity effects causes not only changes in energy level but alter the density of states. Superconductivity emerges from pairing of electrons near the Fermi energy and it is important to know how the density of states at the Fermi energy *N*(0) changes with decoration.

### Fitting of the seven-band tight binding model to DFT

**Figure 3:**
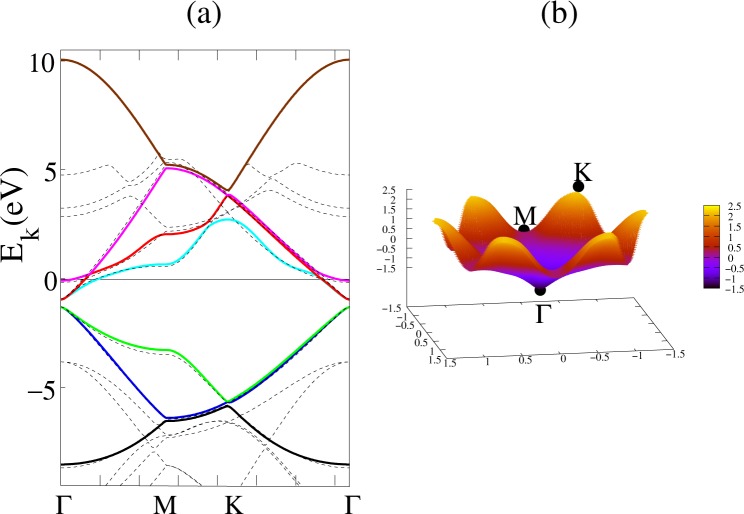
The left panel provides the band structure of lithium decorated graphene. The dashed lines indicate the DFT bands, while the fitted bands are shown in color. The Fermi energy set to zero at *μ*_0_ = 0.4 *eV*. A small gap, *E*_*g*_ = 0.36 *eV* is opened at the Γ point around −1.12 eV. The right panel provides a surface plot of the relatively flat band of LiC_6_. *d*-wave pairing dominates due to electrons in the valleys around saddle points at *M*.

The seven band tight binding model of LiC_6_ was fit to the DFT band structure, with results illustrated in Fig. 3. In the graphene layer shown in Fig. 1(a,c), *A*_1_ subsite chosen as central site labeled by 0 and *B*_1_ subsite in adjacent hexagon considered as second neighbor while just slightly longer than the first neighbors atoms *B*_2_ and *B*_3_ in same hexagon, this neighbor labeled by *n* = 2 and so on the next neighbors are labeled. In Fig. 1(a), the big dashed hexagon included up to nine neighbors but for the pristine graphene it is surrounded by five neighbors. C-C hopping from 0-subsite to *n*th neighbor has been shown by ${t}_{0n}^{CC}$. In-plane Li-Li hopping, ${t}_{0m}^{LiLi}$ obtained up to *m* = 4 neighbors. Li to C hopping integrals are very small with respect to those of C-C and Li-Li, so we keep only the near neighbor Li-C hopping amplitude.

Since Li is small with respect to alkaline earths such as Ca, the pristine band structure is less affected by decoration by lithium than by calcium, as can be seen in Fig. 2 of ref.^15^. The fitted hopping amplitudes and on-site energies are presented in Table 1. Note that by comparing band structure of LiC_6_ with pristine graphene in ref.^23^, it is observed that Li decoration only slightly changes the pristine graphene band structure. These changes are due to electron transfer from Li to graphene, which changes the pristine on site *ε*_*pristine*_ = 0 to *ε*_*A*_ = *ε*_*B*_ = *ε*_*c*_.

**Table 1:** The C-C hopping parameters (eV) for LiC_6_ are denoted by ${t}_{0n}^{CC}$ where the index *n* indicates *n*-th neighbours.

*n*	0	1	2	3	4	5	6	7	8	9
${t}_{0n}^{CC}$	*ε*_*c*_ = −0.77	*t*_1_ = 2.93	${t}_{1}^{\prime} =0.94{t}_{1}$	*t*_2_ = −0.22	${t}_{2}^{\prime} =0.94{t}_{2}$	*t*_3_ = 0.28	${t}_{3}^{\prime} \approx {t}_{3}$	*t*_4_ = −0.03	${t}_{4}^{\prime} \approx {t}_{4}$	*t*5 = −0.05
*m*	0	1	2	3	4					
${t}_{0m}^{LiLi}$	*εLi* = 1.1	−0.30	0.09	0.04	−0.03					
${t}_{0m}^{LiC}$	—	0.30								

In the Li plane, Li-Li hopping parameters are denoted by ${t}_{0m}^{LiLi}$ where *m* is *m*-th Li neighbor of central Li. The Li-C hopping parameter is ${t}_{0m}^{LiC}$.

## Superconducting Pairing and States

### Bogoliubov-de Gennes Transformation

**Figure 4:**
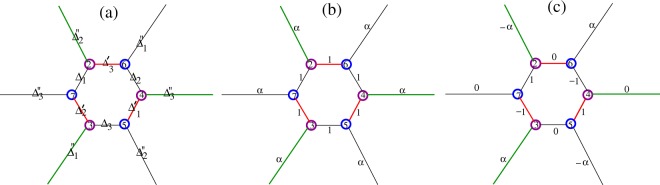
(**a**) Designation of the pairing amplitudes considered in this study, which cover all nearest neighbor pairing possibilities denoted by Δ_*n*〈*ij*〉_, ${{\rm{\Delta }}}_{n\langle ij\rangle }^{^{\prime} }$ and ${{\rm{\Delta }}}_{n\langle ij\rangle }^{^{\prime\prime} }$ where subscript 〈*ij*〉 has been dropped for brevity. (**b**) Shows the pairing amplitude for ${{\rm{\Phi }}}_{S}^{+}$ phase with *α* ≈ 0.6 and for ${{\rm{\Phi }}}_{S}^{-}$ phase with *α* ≈ −3.4. Both phases broken two band graphene symmetry as can be seen by comparing symmetries along different bonds in seven atoms unit cell and two bands unit cell where its Bravais lattice points are labeled by 5, 6 and 7. (**c**) Shows the pairing amplitude ${{\rm{\Phi }}}_{{d}_{xy}}^{+}$ where *α* ≈ 1 and ${{\rm{\Phi }}}_{{d}_{xy}}^{-}$ where *α* ≈ −2. The first phase approximately preserves two band graphene symmetry while the others arise from broken symmetry.

LiC_6_ presents a multiband system in which three bands cross the Fermi level. We presume singlet pairing that can be both intraband and interband in nature. We adopt a local viewpoint in which pairing occurs between electrons on carbon atoms. Seven hybridized Li-C orbitals, support nine possible bond pairing amplitudes in real space. Figure 4(a) illustrates all the nearest neighbour order parameters possibilities. Leaving the analytical derivation details to Supplementary Materials Appendices D and E, the quasiparticle energies are obtained by Bogoliubov-de Gennes unitary transformation in the seven band space,

7 $${E}_{m,s}^{Q}(\overrightarrow{k})=s({E}_{m}(\overrightarrow{k})+\sum _{i=1}^{7}\,\frac{{|{{\rm{\Delta }}}_{mi}(\overrightarrow{k})|}^{2}}{{E}_{m}(\overrightarrow{k})+{E}_{i}(\overrightarrow{k})})\,s=\pm \,1$$

in which *s* = 1 is for particles and *s* = −1 for holes, and *E*_*m*_ are the normal state eigenvalues. The $\overrightarrow{k}$-dependent gap $|{{\rm{\Delta }}}_{mi}(\overrightarrow{k}{)|}^{2}$ in the spectrum are expressed as

8 $${{\rm{\Delta }}}_{mn}(\overrightarrow{k})=\sum _{\alpha =1}^{9}\,{{\rm{\Omega }}}_{mn}^{\alpha }(\overrightarrow{k}){{\rm{\Delta }}}^{\alpha }$$

in which *m* and *n* are band indexes. The band pair order parameter ${{\rm{\Delta }}}_{mn}(\overrightarrow{k})$ denotes pairing between electrons in the *m*-th and *n*-th bands in LiC_6_. Also, $({{\rm{\Delta }}}^{1},{{\rm{\Delta }}}^{2},{{\rm{\Delta }}}^{3})=({{\rm{\Delta }}}_{1}^{^{\prime\prime} },{{\rm{\Delta }}}_{2}^{^{\prime\prime} },{{\rm{\Delta }}}_{3}^{^{\prime\prime} })$; $({{\rm{\Delta }}}^{4},{{\rm{\Delta }}}^{5},{{\rm{\Delta }}}^{6})=({{\rm{\Delta }}}_{1},{{\rm{\Delta }}}_{2},{{\rm{\Delta }}}_{3})$; $({{\rm{\Delta }}}^{7},{{\rm{\Delta }}}^{8},{{\rm{\Delta }}}^{9})=({{\rm{\Delta }}}_{1}^{^{\prime} },{{\rm{\Delta }}}_{2}^{^{\prime} },{{\rm{\Delta }}}_{3}^{^{\prime} })$ are shown in Fig. 4(a), and

9 $$\begin{array}{c}{{\rm{\Omega }}}_{ij}^{1}(\overrightarrow{k})={{\mathscr{C}}}_{1}^{\ast }({E}_{i}){{\mathscr{C}}}_{4}({E}_{j}){e}^{i\overrightarrow{k}.{\overrightarrow{\tau }}_{1}}+{{\mathscr{C}}}_{4}^{\ast }({E}_{i}){{\mathscr{C}}}_{1}({E}_{j}){e}^{-i\overrightarrow{k}.{\overrightarrow{\tau }}_{1}}\\ {{\rm{\Omega }}}_{ij}^{2}(\overrightarrow{k})={C}_{3}^{\ast }({E}_{i}){{\mathscr{C}}}_{6}({E}_{j}){e}^{i\overrightarrow{k}.{\overrightarrow{\tau }}_{2}}+{{\mathscr{C}}}_{6}^{\ast }({E}_{i}){{\mathscr{C}}}_{3}({E}_{j}){e}^{-i\overrightarrow{k}.{\overrightarrow{\tau }}_{2}}\\ {{\rm{\Omega }}}_{ij}^{3}(\overrightarrow{k})={{\mathscr{C}}}_{2}^{\ast }({E}_{i}){{\mathscr{C}}}_{5}({E}_{j}){e}^{i\overrightarrow{k}.{\overrightarrow{\tau }}_{3}}+{{\mathscr{C}}}_{5}^{\ast }({E}_{i}){{\mathscr{C}}}_{2}({E}_{j}){e}^{-i\overrightarrow{k}.{\overrightarrow{\tau }}_{3}}\\ {{\rm{\Omega }}}_{ij}^{4}(\overrightarrow{k})={{\mathscr{C}}}_{2}^{\ast }({E}_{i}){{\mathscr{C}}}_{6}({E}_{j}){e}^{i\overrightarrow{k}.{\overrightarrow{\delta }}_{1}}+{{\mathscr{C}}}_{6}^{\ast }({E}_{i}){{\mathscr{C}}}_{2}({E}_{j}){e}^{-i\overrightarrow{k}.{\overrightarrow{\delta }}_{1}}\\ {{\rm{\Omega }}}_{ij}^{5}(\overrightarrow{k})={{\mathscr{C}}}_{1}^{\ast }({E}_{i}){{\mathscr{C}}}_{5}({E}_{j}){e}^{i\overrightarrow{k}.{\overrightarrow{\delta }}_{2}}+{{\mathscr{C}}}_{5}^{\ast }({E}_{i}){{\mathscr{C}}}_{1}({E}_{j}){e}^{-i\overrightarrow{k}.{\overrightarrow{\delta }}_{2}}\\ {{\rm{\Omega }}}_{ij}^{6}(\overrightarrow{k})={{\mathscr{C}}}_{3}^{\ast }({E}_{i}){{\mathscr{C}}}_{4}({E}_{j}){e}^{i\overrightarrow{k}.{\overrightarrow{\delta }}_{3}}+{{\mathscr{C}}}_{4}^{\ast }({E}_{i}){{\mathscr{C}}}_{3}({E}_{j}){e}^{-i\overrightarrow{k}.{\overrightarrow{\delta }}_{3}}\\ {{\rm{\Omega }}}_{ij}^{7}(\overrightarrow{k})={{\mathscr{C}}}_{3}^{\ast }({E}_{i}){{\mathscr{C}}}_{5}({E}_{j}){e}^{i\overrightarrow{k}.{\overrightarrow{\delta }}_{1}}+{{\mathscr{C}}}_{5}^{\ast }({E}_{i}){{\mathscr{C}}}_{3}({E}_{j}){e}^{-i\overrightarrow{k}.{\overrightarrow{\delta }}_{1}}\\ {{\rm{\Omega }}}_{ij}^{8}(\overrightarrow{k})={{\mathscr{C}}}_{2}^{\ast }({E}_{i}){{\mathscr{C}}}_{4}({E}_{j}){e}^{i\overrightarrow{k}.{\overrightarrow{\delta }}_{2}}+{{\mathscr{C}}}_{4}^{\ast }({E}_{i}){{\mathscr{C}}}_{2}({E}_{j}){e}^{-i\overrightarrow{k}.{\overrightarrow{\delta }}_{2}}\\ {{\rm{\Omega }}}_{ij}^{9}(\overrightarrow{k})={{\mathscr{C}}}_{1}^{\ast }({E}_{i}){{\mathscr{C}}}_{6}({E}_{j}){e}^{i\overrightarrow{k}.{\overrightarrow{\delta }}_{3}}+{{\mathscr{C}}}_{6}^{\ast }({E}_{i}){{\mathscr{C}}}_{1}({E}_{j}){e}^{-i\overrightarrow{k}.{\overrightarrow{\delta }}_{3}}.\end{array}$$

where ${{\mathscr{C}}}_{i}({E}_{j})$ are Bloch wave coefficients of the *j*-th band. Possible order parameter symmetries in Eq. 8 are related to symmetries of Bloch wave functions, through ${{\rm{\Omega }}}_{ij}(\overrightarrow{k})$ functions in Eq. 9. In the limiting case of (folded) six band pristine graphene, the symmetry character of different conduction bands along high symmetry lines were provided in Fig. 2. Bloch symmetry character of non-interacting bands specifies the symmetry of the band order parameter.

### Superconducting States

The linearized gap equation, obtained by minimizing the quasiparticle free energy with respect to nearest neighbor order parameters, is

10 $${J}_{\beta }{{\rm{\Delta }}}^{\beta }=-\,\frac{1}{2N}\,\sum _{\alpha =1}^{9}\,[\sum _{\overrightarrow{k}}\,\sum _{n\mathrm{=1}}^{7}\,\sum _{i\mathrm{=1}}^{7}\,\tfrac{\tanh (\tfrac{{E}_{n}^{Q}}{2{k}_{B}T})}{{E}_{n}(\overrightarrow{k})+{E}_{i}(\overrightarrow{k})}({{\rm{\Omega }}}_{ni}^{\alpha }(\overrightarrow{k}){{\rm{\Omega }}}_{ni}^{\ast \beta }(\overrightarrow{k})+{{\rm{\Omega }}}_{ni}^{\beta }(\overrightarrow{k}){{\rm{\Omega }}}_{ni}^{\ast \alpha }(\overrightarrow{k}))]{{\rm{\Delta }}}^{\alpha }\equiv -\,\sum _{\alpha =1}^{9}\,{{\rm{\Gamma }}}_{\beta \alpha }{{\rm{\Delta }}}^{\alpha }\mathrm{.}$$

This equation can be written in matrix form as

11 $$[\begin{array}{lll}{A}_{3\times 3} & {B}_{3\times 3} & {B}_{3\times 3}\\ {B}_{3\times 3} & {C}_{3\times 3} & {D}_{3\times 3}\\ {B}_{3\times 3} & {D}_{3\times 3} & {C}_{3\times 3}\end{array}]\,(\begin{array}{l}{g}_{1}{V}_{1}\\ {g}_{0}{V}_{2}\\ {g}_{0}{V}_{3}\end{array})=-\,(\begin{array}{l}{V}_{1}\\ {V}_{2}\\ {V}_{3}\end{array})$$

where

12 $$\begin{array}{c}{A}_{3\times 3}=[\begin{array}{lll}{{\rm{\Gamma }}}_{11} & {{\rm{\Gamma }}}_{12} & {{\rm{\Gamma }}}_{12}\\ {{\rm{\Gamma }}}_{12} & {{\rm{\Gamma }}}_{11} & {{\rm{\Gamma }}}_{12}\\ {{\rm{\Gamma }}}_{12} & {{\rm{\Gamma }}}_{12} & {{\rm{\Gamma }}}_{11}\end{array}],{C}_{3\times 3}=[\begin{array}{lll}{{\rm{\Gamma }}}_{44} & {{\rm{\Gamma }}}_{45} & {{\rm{\Gamma }}}_{45}\\ {{\rm{\Gamma }}}_{45} & {{\rm{\Gamma }}}_{44} & {{\rm{\Gamma }}}_{45}\\ {{\rm{\Gamma }}}_{45} & {{\rm{\Gamma }}}_{45} & {{\rm{\Gamma }}}_{44}\end{array}],\\ {B}_{3\times 3}=[\begin{array}{lll}{{\rm{\Gamma }}}_{14} & {{\rm{\Gamma }}}_{15} & {{\rm{\Gamma }}}_{15}\\ {{\rm{\Gamma }}}_{15} & {{\rm{\Gamma }}}_{14} & {{\rm{\Gamma }}}_{15}\\ {{\rm{\Gamma }}}_{15} & {{\rm{\Gamma }}}_{15} & {{\rm{\Gamma }}}_{14}\end{array}],{D}_{3\times 3}=[\begin{array}{lll}{{\rm{\Gamma }}}_{47} & {{\rm{\Gamma }}}_{48} & {{\rm{\Gamma }}}_{48}\\ {{\rm{\Gamma }}}_{48} & {{\rm{\Gamma }}}_{47} & {{\rm{\Gamma }}}_{48}\\ {{\rm{\Gamma }}}_{48} & {{\rm{\Gamma }}}_{48} & {{\rm{\Gamma }}}_{47}\end{array}]\end{array}$$

and ${g}_{1}{V}_{1}={({{\rm{\Delta }}}_{1}^{^{\prime\prime} }{{\rm{\Delta }}}_{2}^{^{\prime\prime} }{{\rm{\Delta }}}_{3}^{^{\prime\prime} })}^{T}$, ${g}_{0}{V}_{2}={({{\rm{\Delta }}}_{1}{{\rm{\Delta }}}_{2}{{\rm{\Delta }}}_{3})}^{T}$ and ${g}_{0}{V}_{3}={({{\rm{\Delta }}}_{1}^{^{\prime} }{{\rm{\Delta }}}_{2}^{^{\prime} }{{\rm{\Delta }}}_{3}^{^{\prime} })}^{T}$; the subscripts 〈*ij*〉 has been dropped for brevity. The *A*_3×3_, *B*_3×3_, *C*_3×3_, and *D*_3×3_ matrices, given by Eq. 12, have identical structures, hence they share eigenvectors: ${V}_{s}^{T}=\mathrm{(1}\,1\,\mathrm{1)}$, ${V}_{{d}_{xy}}^{T}=(1\,-\,1\,0)$, and ${V}_{{d}_{{x}^{2}-{y}^{2}}}^{T}=\mathrm{(1}\,1\,-\,\mathrm{2)}$, where the latter two are degenerate. Their eigenvalues, in obvious notation, are

13 $$\begin{array}{llll}{a}_{s}={{\rm{\Gamma }}}_{11}+2{{\rm{\Gamma }}}_{12}, & {b}_{s}={{\rm{\Gamma }}}_{14}+2{{\rm{\Gamma }}}_{15}, & {c}_{s}={{\rm{\Gamma }}}_{44}+2{{\rm{\Gamma }}}_{45}, & {d}_{s}={{\rm{\Gamma }}}_{47}+2{{\rm{\Gamma }}}_{48}\\ {a}_{d}={{\rm{\Gamma }}}_{11}-{{\rm{\Gamma }}}_{12}, & {b}_{d}={{\rm{\Gamma }}}_{14}-{{\rm{\Gamma }}}_{15}, & {c}_{d}={{\rm{\Gamma }}}_{44}-{{\rm{\Gamma }}}_{45}, & {d}_{d}={{\rm{\Gamma }}}_{47}-{{\rm{\Gamma }}}_{48}.\end{array}$$

For folded six band pure graphene *g*_0_ = *g*_1_, the Bloch wave coefficients appearing in Eq. 9 can be replaced from Eq. B.7 to show that ${{\rm{\Omega }}}_{ij}^{1}(\overrightarrow{k})={{\rm{\Omega }}}_{ji}^{4}(\overrightarrow{k})={{\rm{\Omega }}}_{ij}^{7}(\overrightarrow{k})$ and similarly relations for other elements, hence *C*_3×3_ = *A*_3×3_ and *D*_3×3_ = *B*_3×3_. Eq. 11 takes the more symmetric form

14 $$[\begin{array}{lll}{A}_{3\times 3} & {B}_{3\times 3} & {B}_{3\times 3}\\ {B}_{3\times 3} & {A}_{3\times 3} & {B}_{3\times 3}\\ {B}_{3\times 3} & {B}_{3\times 3} & {A}_{3\times 3}\end{array}]\,(\begin{array}{l}{V}_{1}\\ {V}_{2}\\ {V}_{3}\end{array})=-\,\frac{1}{{g}_{0}}(\begin{array}{l}{V}_{1}\\ {V}_{2}\\ {V}_{3}\end{array})$$

For the case *V*_1_ = *V*_2_ = *V*_3_ = *V*_*sy*_ where *sy* subscripts indicates each of the *s*, *d*_*xy*_ or ${d}_{{x}^{2}-{y}^{2}}$ symmetry, the six band gap Eq. 14 reduces to $(A+2B){V}_{sy}=-\,\frac{1}{{g}_{0}}{V}_{sy}$, i.e. the linearized gap equation of the two band model of pristine graphene in ref.^12^. These three solutions preserve symmetry of the two band unit cell as illustrated in Fig. 4(b,c). In addition to these three states, there are six more non-orthogonal solutions Φ_0*n*_ = (*V*_*sy*_ 0 −*V*_*sy*_) and Φ_1*n*_ = (*V*_*sy*_ −*V*_*sy*_ 0) that break symmetries of pristine graphene two band model. Inserting these solutions into Eq. 14 leads to a new two band gap equation, $(A-B){V}_{sy}=-\,\frac{1}{{g}_{0}}{V}_{sy}$, which is unphysical because of an unreachably high energy pairing potential *g*_0_. In the following section the superconducting gap equation has been solved for *LiC*_6_ and it is demonstrated how Li-C coupling influences superconducting phases.

### Nine Superconducting Phases

**Figure 5:**
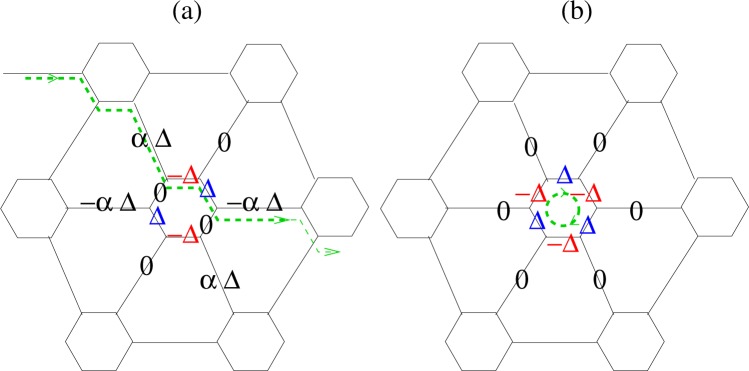
Schematic diagram illustrating Cooper pair propagation for (**a**) ${{\rm{\Phi }}}_{xy}^{J}$ along a chain as shown by the green dashed line and arrow, and (**b**) localized “island” pairs for Φ_*f*_.

Self-consistent solutions of the gap equation Eq. 11 can be obtained analytically. There are three superconducting states with island character (discussed in more detail below) that can be expressed in compact form as

15 $${[{{\rm{\Phi }}}_{n}]}^{T}=[0\,{V}_{sy}\,-\,{V}_{sy}],\,{J}_{sy}^{0}={c}_{sy}-{d}_{sy}$$

where *V*_*sy*_ refers to one of the *V*_*s*_, ${V}_{{d}_{xy}}$ or ${V}_{{d}_{{x}^{2}-{y}^{2}}}$-wave symmetries. Pairing in these phases cannot propagate, as may be pictured in Fig. 5. The other six superconducting states of Eq. 11 have the explicit form

16 $${[{{\rm{\Phi }}}_{n}]}^{T}=[{\alpha }_{sy}^{\pm }{V}_{sy}\,{V}_{sy}\,{V}_{sy}]$$

corresponding to the interaction potential is ${g}_{0}=\frac{1}{{J}_{sy}}$ wherein

17 $${\alpha }_{sy}^{\pm }=\frac{{J}_{sy}^{\pm }-{c}_{sy}-{d}_{sy}}{{b}_{sy}},\,{J}_{sy}^{\pm }=\frac{1}{2}(\kappa {a}_{sy}+{c}_{sy}+{d}_{sy}\pm \sqrt{8\kappa {b}_{sy}^{2}+{[{c}_{sy}+{d}_{sy}-\kappa {a}_{sy}]}^{2}})$$

In these expressions ${J}_{d(s)}^{\pm }$ and ${\alpha }_{d(s)}^{\pm }$ are obtained from Eq. 17 by substituting *a*_*sy*_, *b*_*sy*_, *c*_*sy*_, and *d*_*sy*_ by *a*_*d*(*s*)_, *b*_*d*(*s*)_, *c*_*d*(*s*)_, and *d*_*d*(*s*)_ respectively.

By comparing the gap equations introduced in Eqs 11 and 14 the gap equation symmetry reduction of decorated graphene with respect to folded but pristine graphene becomes clear. This symmetry reduction results in an *α*_*sy*_ coefficient appearing in the pairing amplitudes of stretched bonds as shown in Eq. 16 and Fig. 4. We refer to these symmetry reduction phases as “distorted phases”.

The six bands of pristine graphene support nine pairing amplitudes while in the two band model there are three possible pairing amplitudes along three different bonds. These two notions can be mapped onto each other only if *α*_*sy*_ = 1 as illustrated in Fig. 4(b,c). Therefor the three island superconducting phases given by Eq. 15 in the special case of pristine cannot be mapped onto the two band model. These three phases are unphysical even in the case of decorated graphene because the Cooper pairs in these phases require a large pairing potential. In the special case of pristine graphene in which *κ* = 1, *a*_*sy*_ = *c*_*cy*_ and *b*_*sy*_ = *d*_*cy*_ from Eq. 17, and it follows that if *b*_*sy*_ > 0 then ${\alpha }_{sy}^{+}=1$ and ${\alpha }_{sy}^{-}=-\,2$. Also ${g}_{0}^{+} < {g}_{0}^{-}$ so in this case (+) sign preserves the two band model while the (−) sign phases are unphysical. Numerical calculation shows that ${b}_{sy}^{+} > 0$. These superconducting states can be categorized into three groups according to their corresponding pairing potential.

**3 electron pairing states with island character and very high pairing potential;** (**unphysical solutions**)

18 $$\begin{array}{rcl}{{\rm{\Phi }}}_{{p}_{x}} & = & \frac{1}{2}{[(000)(1-10)(-110)]}^{T},\,{g}_{0}=\frac{1}{{c}_{d}-{d}_{d}},\\ {{\rm{\Phi }}}_{{p}_{y}} & = & \frac{1}{2\sqrt{3}}{[(000)(11-2)(-1-12)]}^{T},\,{g}_{0}=\frac{1}{{c}_{d}-{d}_{d}},\\ {{\rm{\Phi }}}_{f} & = & \frac{1}{\sqrt{6}}{[(000)(111)(-1-1-1)]}^{T},\,{g}_{0}=\frac{1}{{c}_{s}-{d}_{s}}.\end{array}$$

**3 states with higher electron pairing potential;** (**unphysical solutions**)

19 $$\begin{array}{rcl}{{\rm{\Phi }}}_{{d}_{{x}^{2}-{y}^{2}}}^{-} & = & {(6{({\alpha }_{d}^{-})}^{2}+12)}^{-\frac{1}{2}}{[{\alpha }_{d}^{-}(11-2)(11-2)(11-2)]}^{T},\,{g}_{0}=\frac{1}{{J}_{d}^{-}}\\ {{\rm{\Phi }}}_{{d}_{xy}}^{-} & = & {(2{({\alpha }_{d}^{-})}^{2}+4)}^{-\frac{1}{2}}{[{\alpha }_{d}^{-}(1-10)(1-10)(1-10)]}^{T},\,{g}_{0}=\frac{1}{{J}_{d}^{-}}\\ {{\rm{\Phi }}}_{s}^{-} & = & {(3{({\alpha }_{s}^{-})}^{2}+\mathrm{\ \ 6})}^{-\frac{1}{2}}{[{\alpha }_{s}^{-}(111)(111)(111)]}^{T},\,{g}_{0}=\frac{1}{{J}_{s}^{-}}\end{array}$$

**3 states with lower electron pairing potential;** (**physical solutions**)

20 $$\begin{array}{rcl}{{\rm{\Phi }}}_{{d}_{{x}^{2}-{y}^{2}}}^{+} & = & {(6{({\alpha }_{d}^{+})}^{2}+12)}^{-\frac{1}{2}}\,{[{\alpha }_{d}^{+}(11-2)(11-2)(11-2)]}^{T},{g}_{0}=\frac{1}{{J}_{d}^{+}}\\ {{\rm{\Phi }}}_{{d}_{xy}}^{+} & = & {(2{({\alpha }_{d}^{+})}^{2}+4)}^{-\frac{1}{2}}\,{[{\alpha }_{d}^{+}(1-10)(1-10)(1-10)]}^{T},{g}_{0}=\frac{1}{{J}_{d}^{+}}\\ {{\rm{\Phi }}}_{s}^{+} & = & {(3{({\alpha }_{s}^{+})}^{2}+\mathrm{\ \ 6})}^{-\frac{1}{2}}\,{[{\alpha }_{s}^{+}(111)(111)(111)]}^{T},{g}_{0}=\frac{1}{{J}_{s}^{+}}.\end{array}$$

All states are orthogonal except those with same subscript, viz. ${{\rm{\Phi }}}_{s}^{-}$ and ${{\rm{\Phi }}}_{s}^{+}$. Such solutions are orthogonal if *κ* = 1, *i*.*e*. *g*_1_ = *g*_0_. Only for this case the matrix gap equation becomes Hermitian, then band order parameters takes the following form in terms of the band Green function and *g*_0_,

21 $${{\rm{\Delta }}}_{ij}(\overrightarrow{k})={g}_{0}\langle {d}_{i}^{\uparrow }(\overrightarrow{k}){d}_{j}^{\downarrow }(\overrightarrow{k})\rangle .$$

Here ${\hat{d}}_{i}^{\sigma }(\overrightarrow{k})={\sum }_{m=1}^{7}\,{{\mathscr{C}}}_{m}^{\ast }({E}_{i}(\overrightarrow{k})){\hat{c}}_{m}^{\sigma }(\overrightarrow{k})$ annihilates an electron with spin *σ* in the *i*th band with energy ${E}_{i}(\overrightarrow{k})$. Although it is assumed that *g*_1_ = *g*_0_ but deviation from pristine leads to distortion of Green’s functions $\langle {\hat{c}}_{i\alpha }^{\sigma }{\hat{c}}_{j\beta }^{\sigma }\rangle$ along different bonds.

**Figure 6:**
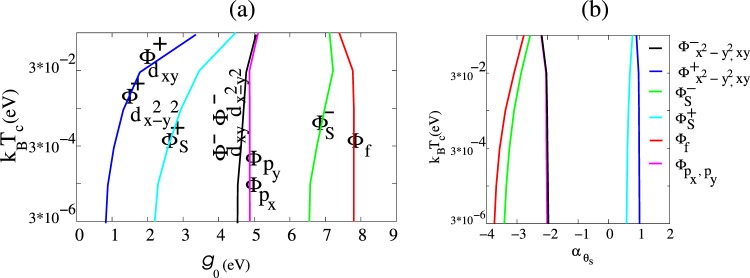
(**a**) This phase diagram illustrates the relation between *T*_*c*_ and the pairing potential *g*_0_ for Li*C*_6_ in which *μ*_0_ = 0. Panel (b) shows *T*_*c*_ in terms of *α*_*sy*_

Phases ${{\rm{\Phi }}}_{{d}_{{x}^{2}-{y}^{2}}}^{-}$ and ${{\rm{\Phi }}}_{{d}_{xy}}^{-}$ are degenerate with eigenvalue ${J}_{{d}_{{x}^{2}-{y}^{2}}}^{-}={J}_{{d}_{xy}}^{-}={J}_{d}^{-}$, and similarly ${{\rm{\Phi }}}_{{d}_{{x}^{2}-{y}^{2}}}^{+}$ and ${{\rm{\Phi }}}_{{d}_{xy}}^{+}$ with eigenvalue ${J}_{d}^{+}$. For Li decorated graphene, numerical calculation shows ${J}_{sy}^{+} > {J}_{sy}^{-}$, so *g*_0_ in the (+) states is lower than *g*_0_ in the (−) states hence pairing in this modes are dominant. From Eqs 19 and 20 we observe that probability amplitudes for pairing on different bonds in real space differ for the various states. For the long C-C bonds the probability is proportional to ${({\alpha }_{sy}^{\pm })}^{2}$ while for the others is unity. Numerical results are shown in Fig. 6.

## Discussion and Relation To Previous Work

The possibility of a superconductivity state in metal decorated graphene has been suggested theoretically by a few groups^9,12,15^. Some have suggested phonon-mediated superconductivity in single layer graphene. Most prominently, Profeta *et al*.^15^ calculated on the basis of density functional theory for superconductors that decoration by electron donating atoms such as Ca and Li will make single layer graphene superconducting, up to 8 K for the case of Li. The *ab initio* anisotropic Migdal-Eliashberg formalism was used by Zheng and Margine^24^, who predicted a single anisotropic superconducting gap with critical temperature *T*_*c*_ = 5.1–7.6 *K*, in surprisingly good agreement with experimental reported superconductivity around 6 K in *LiC*_6_^5^.

Using a phenomenological microscopic Hamiltonian in a nearest-neighbor tight-binding approximation, possible superconducting phases of pristine graphene have been discussed by Uchoa and Castro-Neto^9^ and also by Black-Schaffer and Doniach^12^. The possibility of a singlet *p* + *ip* phase pairing near the Dirac points between nearest neighbors subsites were suggested by Uchoa and Castro-Neto^9^. They worked in terms of a plasmon mediated mechanism for metal coated graphene, and discussed the conditions under which attractive electron-electron interaction can be mediated by plasmons.

Singlet superconducting gap phases of pristine graphene have been proposed and discussed by Black-Schaffer and Doniach^12^. For the nearest neighbors pairing amplitudes ${{\rm{\Delta }}}_{\langle iAjB\rangle }={{\rm{\Delta }}}_{iA,iA+{\overrightarrow{\delta }}_{j}}$ where ${\overrightarrow{\delta }}_{j}$ are the vectors that connects the *iA* site to its three nearest neighbors, it was observed that there are three states that minimize the free energy in various regimes of the parameters, which here have been denoted by *V*_*s*_ = (1, 1, 1)^*T*^, ${V}_{{d}_{{x}^{2}-{y}^{2}}}={(2,-1,-1)}^{T}$, and ${V}_{{d}_{xy}}={(0,-1,1)}^{T}$. Pairing symmetries *d*_*xy*_ and ${d}_{{x}^{2}-{y}^{2}}$ are degenerate, and only the linear combination of ${d}_{{x}^{2}-{y}^{2}}+i{d}_{xy}\equiv d+id$ preserves the graphene band symmetry. Depending on the position of the Fermi energy with respect to Dirac points, *d* + *id* or *s* states tend to dominate. Their numerical calculation showed that *d*-wave solutions will always be favored for electron or hole doping in the regime $0 < {\bar{n}}_{c} < 0.4$ where doping is defined by ${\bar{n}}_{\alpha }=\langle {\hat{c}}_{i\alpha }^{\dagger }{\hat{c}}_{i\alpha }\rangle -1$. In this regime, superconductivity can emerge from electronic correlation effects. Near the van Hove singularity at the saddle point *M* corresponding to 3/8 and 5/8 fillings i.e. ${\bar{n}}_{c}=0.25$, it was suggested that chiral *d* + *id* superconductivity, which breaks time-reversal symmetry, can be stabilized. In this regime *d*-wave superconductivity may arise from repulsive electron-electron interaction^11^.

Although doping by a gate voltage is normally considered to change only the chemical potential but not the band structure, gating cannot be expected to push the Fermi energy to the van Hove singularity without altering the band dispersion. The most likely way to do this is by decoration with electropositive atoms, which has been our focus. We note that doping is essential, when graphene decorated, in addition to the expected charge migration from the decorating atoms to the graphene sheet, it is then necessary the interlayer state is partially occupied to induce superconductivity as happens in GICs. Hybridization of interlayer *s*-band and graphene *π* bands changes the graphene band structure. The *s* orbitals of Ca have more overlap with C orbitals than Li and lead to stronger and longer range interactions as well as increasing the doping level, effects that become detrimental to superconductivity. For this reason our emphasis here is on the Li decorated graphene.

We review some of our main points. When graphene is decorated by Li, electron transfer from Li atoms to C contracts the Li-C distance and reduces the C-C bond lengths in the Li-centered hexagon. In this kekulé -type structure, hopping amplitude symmetries of all C-C neighbors are broken (our “shrunken graphene”). This model allows study of multiband effects on the superconducting phase diagram. To gain insight into our model, solutions of superconducting gap equation in both cases of folded bands otherwise pristine C_6_ and the usual two band model of C_2_ were compared. These two viewpoints coincide if the same pairing paradigms are considered. For pristine graphene with its two site cell, in real space picture electrons can pair with near neighbors in three inequivalent directions, ${{\rm{\Delta }}}_{i,i+\overrightarrow{\delta }}={V}_{sy}={({{\rm{\Delta }}}_{1}{{\rm{\Delta }}}_{2}{{\rm{\Delta }}}_{3})}^{T}$ which must respect honeycomb symmetries. The *V*_*sy*_ quantities are the three vectors that belong to the irreducible representation of crystal point group D_6*h*_ i.e. ${V}_{sy}^{T}$ = (1, 1, 1), (−1, 1, 0) and (2, −1, −1) for which the *sy* subscript stands for symmetries *s*, *d*_*xy*_ and ${d}_{{x}^{2}-{y}^{2}}$. Permutation of *s*-wave solution (1, 1, 1) along three different bonds constructs just one state while permutation of d_*xy*_ solution (−1, 1, 0) up to a minus sign constructs two nonorthogonal linear independent states viz. (−1, 1, 0) and (−1, 0, 1) which orthogonal linear combination of them are ${{\rm{d}}}_{xy}^{T}$ = (−1, 1, 0) and ${d}_{{x}^{2}-{y}^{2}}^{T}$ = (2 −1 −1).

A similar procedure again can be applied to pristine graphene but now in enlarged six site unit cell. Unit cell of C_6_ includes six carbon subsites and nine different bonds that support nine possible nearest neighbor bond pairing amplitudes as illustrated in Fig. 4 and denoted them by ${{\rm{\Phi }}}_{sy}^{T}=[({{\rm{\Delta }}}_{1}^{^{\prime\prime} },{{\rm{\Delta }}}_{2}^{^{\prime\prime} }\,{{\rm{\Delta }}}_{3}^{^{\prime\prime} })\,({{\rm{\Delta }}}^{1},{{\rm{\Delta }}}^{2},{{\rm{\Delta }}}^{3})\,({{\rm{\Delta }}}_{1}^{^{\prime} },{{\rm{\Delta }}}_{2}^{^{\prime} }\,{{\rm{\Delta }}}_{3}^{^{\prime} })]$. The gap equation is a 9 × 9 matrix equation given by Eq. 14. The folded bands supercell include three vertices numbered 5, 6, 7, and nine bonds as shown in Fig. 4(a). There are nine orthogonal solutions that preserve symmetries of this supercell. One of these configurations has s-wave symmetry (1, 1, 1, 1, 1, 1, 1, 1, 1) the other eight solutions are constructed by all possible permutations of (−1, 1, 0) along these bonds that preserve our supercell symmetry. There are only three solutions which can preserve symmetry of both two and six atoms cells simultaneously which they are of the form ${{\rm{\Phi }}}_{sy}^{+}={({V}_{sy}{V}_{sy}{V}_{sy})}^{T}$ as illustrated in Fig. 4. For these solutions, the folded 9 × 9 gap equation reduces to 3 × 3 gap equations of ordinary pristine graphene. The Cooper pair formation energy for these three modes are significantly less than the other six phases which are not reducible to the two band model.

In fact reduction of symmetry leads to increasing of the system free energy. After the orthogonalization procedure, one obtains three solutions Φ_*f*_, ${{\rm{\Phi }}}_{{p}_{x}}$ and ${{\rm{\Phi }}}_{{p}_{y}}$, of the form ${{\rm{\Phi }}}_{sy}^{0}={\mathrm{(0}{V}_{sy}-{V}_{sy})}^{T}$. These phases have been designated as island phases, as illustrated in Fig. 5(b) for Φ_*f*_, within which a pairing amplitude is localized within island hexagons and cannot propagate. For these island phases, numerical calculation of the electron pair potential energy *g*_0_ shows that *g*_0_ is large. This kind of solutions is a consequence of the six atom basis and does not appear for the two atom basis. Also, there are three solutions of the form ${{\rm{\Phi }}}_{sy}^{-}=(\,-\,2{V}_{sy}\,{V}_{sy}\,{V}_{sy})$ which also break symmetry of two atom cell. For these reasons, in association with the normal state band structure of graphene, we concentrate on superconductivity in the three ${{\rm{\Phi }}}_{sy}^{+}$ symmetry phases.

For pristine graphene C_2_, two normal bands are *E*_±_ = ±*t*_1_|*η*_0_| which fold to six branches in mini-BZ of C_6_ i.e. ${E}_{\gamma }^{\pm }=\pm \,{t}_{1}|{\eta }_{0}|$, ${E}_{\beta }^{\pm }=\pm \,{t}_{1}|{\eta }_{1}|$ and ${E}_{\alpha }^{\pm }=\pm \,{t}_{1}|{\eta }_{2}|$ as shown in figure:eta-k, also Bloch-wave symmetry character of each branch has been distinguished. The Bloch coefficients of the branch labeled by *γ* are of *s*-wave character, ${C}_{{A}_{i}}=(1,1,1)$ and for those labeled as *α* and *β* are of the form *d* ± *id* type, *i*.*e*. ${C}_{{A}_{i}}=(1,{e}^{\pm i\frac{i2\pi }{3}},{e}^{\pm i\frac{i4\pi }{3}})$. Based on Bloch wave character of these branches one can obtain the dominant superconducting phases of pristine graphene in various doping regimes. *d*-wave pairing emerges from the *d*-wave branches of the folded band structure ${E}_{\alpha }^{\pm }$ and ${E}_{\beta }^{\pm }$, while *s*-wave pairing arises from the *s*-wave branch ${E}_{\gamma }^{\pm }$. For folded but otherwise pristine graphene, Fig. 2 illustrates that the lowest conduction band, weakly dispersive along Γ → *M*, is responsible for dominant singlet superconductivity in chiral *d* ± *id* symmetry. Upon electron doping to the critical vHs at ${\bar{n}}_{c}=0.25$, the pairing potential *g*_0_ in the *d* ± *id* phase decreases, beyond which density of states decreases. *g*_0_ increases until a second critical value of doping ${\bar{n}}_{c}=0.4$ at which a phase transition to *s*-wave pairing occurs. Bloch states in higher conduction bands include combinations of *s* and *f* symmetries that favor extended *s* wave pairing. The multiband character is responsible for stabilizing singlet *s* superconductivity at high electron or hole doping.

**Figure 7:**
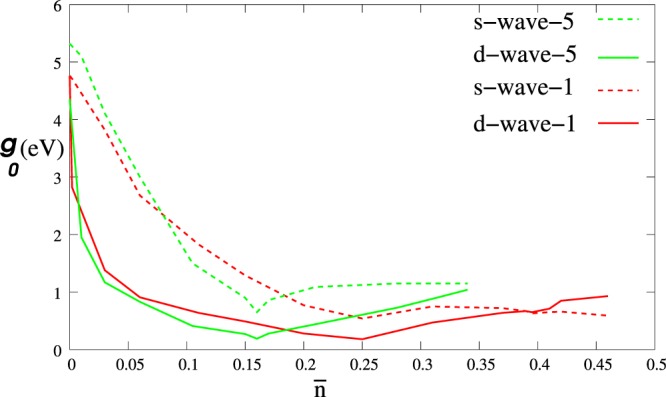
Shows cooper pair interaction *g*_0_ in terms of doping $\bar{n}$ for d and s-wave phases for pristine graphene at T = 0.1 K. The solid (dashed) red line indicates d- wave (s- wave) pairing interaction in first nearest neighbor hopping *t*_1_ = 2.5 *eV* and similarly green line for accurate tight binding model can fit on DFT. For red line at the charge neutrality s- and d- wave are degenerate with *g*_0_ = 4.76 while for full approximation they are not degenerate.

To understand how superconducting phases of graphene can be affected by decoration by Li, one can compare the LiC_6_ gap solutions with those of folded bands C_6_ at the same doping. Numerical results for pristine graphene gap equation performed in the nearest neighbor approximation in ref.^12^ have been extended by applying a more accurate tight binding model fit to the DFT band structure of pristine graphene^23^. Although a quantum critical point for zero doping reported by Black-Schaffer and Doniach^12^ at dimensionless coupling $\frac{{g}_{0}}{t}=1.91$ which *d*- and *s*-wave solutions are degenerate. In the more realistic tight binding model we applied, this degeneracy is not observed at the Γ point, and the *d*-wave solution is dominant. This difference may be consequence of particle-hole symmetry breaking of valence and conduction bands. Also the van Hove singularity at the M point is moved from 0.25 doping for nearest neighbor hopping to 0.16 doping in the accurate model. The phase transition from *d*-wave to *s*-wave is shifted to 0.35 doping instead of the 0.4 doping reported for nearest neighbor hopping^12^. Numerical calculations for this more detailed model are illustrated in Fig. 7.

When graphene is decorated by Li, around 0.68 electron per lithium atom transfers to neighboring C sites, viz. ${\bar{n}}_{c}=0.11$, and the Dirac points folded to Γ move to −1.52 eV. Symmetry breaking of the hopping partially removes degeneracies of band structure of pristine graphene, which leads to creation of the small gap at Γ, with energy ${E}_{g}=2|{t}_{1}-{t}_{1}^{^{\prime} }|=0.36\,eV$. Also two of four-fold degeneracies between valence and conduction bands at the Dirac points are removed. Compression between band structure of decorated graphene and folded pristine graphene at the same doping shows that hybridization of the Li *s* band and C *π* band is small. This means nearest neighbor Li-C hopping is in the range ${t}_{1}^{LiC}$ ~ 0.3–0.5, and further hoppings are negligible.

Li decoration of graphene changes not only the band structure but also the Bloch wave coefficients from those of pristine graphene. While pristine graphene Bloch wave coefficients have pure *s*- or *d*-wave character and their magnitudes are $\overrightarrow{k}$-independent. In the case of LiC_6_ they become mixed and vary with $\overrightarrow{k}$, hence gap equation symmetry is reduced. Because of this symmetry reduction, for the longer C-C bonds, a new coefficient *α*_*sy*_ appears in the pairing amplitudes. In terms of this coefficient we have classified superconducting phase symmetries into three groups. Eqs 18, 19, and 20 present all nine possible pairing phases of LiC_6_. There are three categories of solutions which have not appeared in complete form in the literature. The total of nine phases arise from spatial, and therefore hopping parameter, symmetry breaking.

In the first category Φ_*f*_, ${{\rm{\Phi }}}_{{p}_{x}}$ and ${{\rm{\Phi }}}_{{p}_{y}}$, there is *α*_*sy*_ = 0 identical to that of folded pristine C_6_. For the second category, *α*_*sy*_ (denoted by *α*^−^) is negative, in the case of pristine *α*^−^ = −2 as discussed. These three phases break the two site cell symmetry, and numerical calculation shows that the pairing potential *g*_0_ must be large to realize these phases. For the last category *α*^+^ is positive. Three phases which correspond to *α*^+^ > 0 include ${{\rm{\Phi }}}_{{d}_{{x}^{2}-{y}^{2}}}^{+}$, ${{\rm{\Phi }}}_{{d}_{xy}}^{+}$, and ${{\rm{\Phi }}}_{s}^{+}$, and these have the lowest pairing potentials with respect to the other six phases.

In the limiting case of folded six band pristine graphene ${\alpha }_{{d}_{{x}^{2}-{y}^{2}}}^{+}$, ${\alpha }_{{d}_{xy}}^{+}$, and ${\alpha }_{s}^{+}$ are all equal to unity, which maps the results to the two-band symmetries as it should. But when Li decorated, depending on doping strength viz. *w*_*t*_ and ${t}_{1}^{LiC}$ these coefficients ${\alpha }_{sy}^{+}$ no longer remain unity. The pairing amplitude distortion along longer C-C bonds *α*^+^, for s-wave phase is significant due to its spatial isotropic symmetry. In spite of the pristine nature this phase no longer preserves two band model symmetry. On the other hand, *d*-wave phases are hardly affected by doping and their superconductivity is more persistent against perturbation. The chirality or non-chirality of Cooper pairs in these phases is undetermined, however. As shown in Fig. 6(b), at low temperature ${\alpha }_{s}^{+}\approx 0.6$ for ${{\rm{\Phi }}}_{s}^{+}$, and ${\alpha }_{{d}_{{x}^{2}-{y}^{2}}}^{+}={\alpha }_{{d}_{xy}}^{+}\equiv {\alpha }_{d}^{+}$ is approximately equal to unity and varies little with temperature.

**Figure 8:**
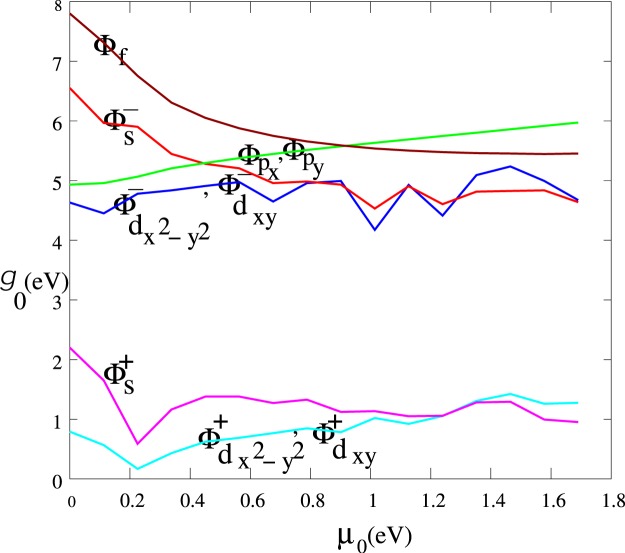
This diagram illustrates interaction potential *g*_0_ in terms of chemical potential *μ*_0_ at *T*_*c*_ = 0.1 K. Upon electron doping to a critical chemical potential *μ*_*o*−*v*_ = 0.22 *eV* (van Hove singularity) for symmetries ${{\rm{\Phi }}}_{{d}_{{x}^{2}-{y}^{2}}}^{+}$, ${{\rm{\Phi }}}_{{d}_{xy}}^{+}$, and ${{\rm{\Phi }}}_{s}^{+}$ the pairing potential decreases, then increases until a second critical value *μ*_*o*−*c*_ = 1.3 eV at which a phase transition to ${{\rm{\Phi }}}_{s}^{+}$ occurs.

At a given critical temperature *T*_*c*_ and chemical potential *μ*_0_, for each of nine possible superconducting phases, Eqs 10, 13 and 17 were evaluated numerically over the BZ of Li*C*_6_ to find the corresponding pairing potential ${g}_{0}=\frac{1}{{J}_{sy}}$ and *α*_*sy*_ coefficient. Smaller *g*_0_ means less Cooper pair formation energy is required. Figure 6(a) provides the phase boundaries for *T*_*c*_ in terms of the pairing potential *g*_0_ for Li*C*_6_ in which *μ*_0_ = 0. For a given transition temperature *T*_*c*_, by changing the chemical potential *μ*_0_ of Li*C*_6_ via gating, one can engineer the pairing potential *g*_0_. Figure 8 gives a *g*_0_-*μ*_0_ phase boundary diagram at *T*_*c*_ = 0.1 K. As illustrated in this figure, similarly to pristine graphene, decoration with Li atoms makes it is possible to change the dominant pairing and to have a symmetry-change phase transition from *d* to “distorted *s*-wave.” Changing *μ*_*o*_ up to *μ*_*o*−*v*_ ≈ 0.22 *eV* so that the distance between the Fermi energy and the saddle points decreases, leads to a decrease in *g*_0_. Continuously increasing *μ*_*o*_ up to 0.5 eV causes *g*_0_ to increase for both *d*-wave and “distorted *s*-wave” pairing, and after that a smooth decrease proceeds. For both symmetries at critical *μ*_*o*−*c*_ = 1.3 eV mixed state exist.

Up to *μ*_*o*−*c*_ = 1.3 eV, the flat band plays a primary role in formation of Cooper pairs with lowest energy. The Bloch wave function of this band consists of *d* and *p* character, therefore Γ_12_, Γ_15_, Γ_45_ and Γ_48_ in Eq. 13 carry minus signs. This makes it evident from Eq. 17 that *d*–wave pairing is dominant. Beyond that, the uneven part of the “flat band” and also upper bands assume a major role. These bands consist of *d*, *p*, *s*, and *f* character Bloch wave functions (as defined in earlier sections) with a significantly low density of states. In this case Γ_12_, Γ_15_, Γ_45_ and Γ_48_ change their sign, hence *s*-wave pairing is favored.

Numerically we have demonstrated that electron pairing *g*_0_ in the limit of pristine graphene is minimal for all dopings. Our calculations indicate that any perturbation of the flat band reduces T_*c*_. The flat band can be perturbed through electron hopping from decorating atoms to carbon sites (${t}_{1}^{LiC}$) or by hopping symmetry breaking index *w*_*t*_. For fixed doping at $\bar{n}$ = 0.11 electron per carbon site and for fixed *w*_*t*_ = 0.94 as obtained for lithium decorated, in a variety of Li-C hopping between 0.3–0.4 eV, numerical calculation doesn’t show significant altering of pair interaction potential *g*_0_ in *s*- and *d*-wave phases. But, as one could see there is not an explicit behavior in a general coupling strength. A result is that a general aspect of superconducting pairing in LiC_6_ and pristine graphene is almost the same in the ${d}_{{x}^{2}-{y}^{2}}$ and *d*_*xy*_ phases due to robustness of the flat band against perturbation.

To summarize, our calculations indicate that *d*-wave phases exist and are dominant symmetry of pairing in both pristine and Li decorated graphene. Pure *s*-wave phase does not appear in LiC_6_, and *s*-wave superconductivity in metal decorated graphene is disfavored because of spatially increased overlap for *s*-wave symmetry. These results show that while degree of doping plays a major role in the graphene superconductivity, perturbation effects of decorating atoms finally determine the phase diagram. Our work also provides a new type of classification of superconducting phases in Li*C*_6_-like nanostructures, and certain aspects of the formalism may be useful in modeling the recently observed superconductivity in magic angle bilayer graphene^3^.

## Electronic supplementary material

Supplementary Materials
