# Supplementary material for: Superconducting Phases in Lithium Decorated Graphene LiC6
Source: Sci Rep. 2018 Sep 14;8:13795. doi: 10.1038/s41598-018-32050-9 (PMC6138680; doi:10.1038/s41598-018-32050-9)
Supplement: Supplementary file 1 — Supplementary Materials [file 41598_2018_32050_MOESM1_ESM.pdf]

Superconducting Phases in Lithium Decorated Graphene LiC_6

Rouhollah Gholami[1], Rostam Moradian[1,2,*], Sina Moradian[3], and Warren E. Pickett[4]

[1]Physics Department, Faculty of Science Razi University, Kermanshah, Iran
[2]Nano science and nano technology research center, Razi University, Kermanshah, Iran
[*]Correspondence and requested materials should be addressed to R. M. [rmoradian@raz.ac.ir]
[3]Department of Electrical and Computer Engineering, University of Central Florida, Orlando, Florida, USA.
[4]Department of Physics UC Davis, One Shield Avenue, Davis, CA 95616, USA.

Supplementary Materials:

APPENDIX A: Accurate Tight Binding Model for Lithium decorated Graphene
APPENDIX B: Uncoupled C_6 Dispersion Relations
APPENDIX C: Coupled Li-C_6 dispersion relations
APPENDIX D: Bogoliubov-de Gennes Transformation
APPENDIX E: Superconducting States

A Accurate Tight Binding Model for Lithium decorated Graphene

The Hamiltonian of non-interacting LiC_6 is

$$\hat{H}_N = -\sum_{i\alpha}\sum_{j\beta,\sigma} t^{\sigma,\sigma}_{i\alpha,j\beta} c^{\dagger}_{i\alpha\sigma} c_{j\beta\sigma} + \sum_{i\alpha,\sigma}(\varepsilon_{i\alpha}-\mu_o)\hat{n}_{i\alpha\sigma}. \tag{A.1}$$

Eq A.1 can be diagonalized in terms of Bloch eigenfunctions of the form

$$\left|\Psi_{\vec{k}}\right\rangle = \frac{1}{\sqrt{N}}\sum_{n=1}^{N}\sum_{\alpha=1}^{7}\mathscr{C}_\alpha e^{i\vec{k}.\vec{r}_{n\alpha}}\left|\phi_{n\alpha}\right\rangle \tag{A.2}$$

in which $\vec{r}_{n\alpha} = \vec{r}_n + \vec{d}_\alpha$ and $\vec{r}_n$ is nth Bravais lattice site vector position and $\vec{d}_\alpha$ is vector position of the α-th subsite with respect to unit cell n. $|\phi_{n\alpha}\rangle = \left|\phi_{n\alpha}(\vec{r}-\vec{r}_n-\vec{d}_\alpha)\right\rangle$ is the atomic π electron ket of atom α of cell n. By inserting Eqs. A.1 and A.2 into $\hat{H}_N(\vec{k})\left|\Psi_{\vec{k}}\right\rangle = E(\vec{k})\left|\Psi_{\vec{k}}\right\rangle$, the equation for the coefficients becomes

$$\sum_{\beta=1}^{7}\varepsilon_{\alpha\beta}(\vec{k})\mathscr{C}_\beta + (\varepsilon_\alpha-\mu_o)\mathscr{C}_\alpha = E(\vec{k})\mathscr{C}_\alpha \quad \text{where} \quad \varepsilon_{\alpha\beta}(\vec{k}) = -\frac{1}{N}\sum_{ij} t^{\sigma\sigma}_{i\alpha j\beta} e^{i\vec{k}.(\vec{r}_{i\alpha}-\vec{r}_{j\beta})}. \tag{A.3}$$

Eq. A.3 can be expressed in following matrix form

$$\left(\begin{array}{c|cc} \varepsilon_{LiLi}(\vec{k})+\varepsilon_{Li}-\mu_o & h_{LiA}(\vec{k}) & h_{LiB}(\vec{k}) \\ \hline h^{\dagger}_{LiA}(\vec{k}) & h_{AA}(\vec{k})+\varepsilon_A-\mu_o & h_{AB}(\vec{k}) \\ h^{\dagger}_{LiB}(\vec{k}) & h_{BA}(\vec{k}) & h_{BB}(\vec{k})+\varepsilon_B-\mu_o \end{array}\right)\left(\begin{array}{c} \mathscr{C}_{Li}(E_i(\vec{k})) \\ \hline \xi_A(E_i(\vec{k})) \\ \xi_B(E_i(\vec{k})) \end{array}\right) = E_i(\vec{k})\left(\begin{array}{c} \mathscr{C}_{Li}(E_i(\vec{k})) \\ \hline \xi_A(E_i(\vec{k})) \\ \xi_B(E_i(\vec{k})) \end{array}\right) \tag{A.4}$$

where

$$\begin{aligned} \varepsilon_{LiLi}(\vec{k}) &= 2t_1^{LiLi}\left(\cos\vec{k}.\vec{\xi}_1+\cos\vec{k}.\vec{\xi}_2+\cos\vec{k}.\vec{\xi}_3\right)+2t_2^{LiLi}\left(\cos\vec{k}.(\vec{\xi}_1-\vec{\xi}_2)+\cos\vec{k}.(\vec{\xi}_1-\vec{\xi}_3)+\cos\vec{k}.(\vec{\xi}_2-\vec{\xi}_3)\right) \\ &+ 2t_3^{LiLi}\left(\cos 2\vec{k}.\vec{\xi}_1+\cos 2\vec{k}.\vec{\xi}_2+\cos 2\vec{k}.\vec{\xi}_3\right)+\dots \end{aligned} \tag{A.5}$$

In Eq. A.4, h-sub-block matrices are C-C or Li-C dispersion matrices and we have $h_{AA} = h^*_{BB}$, $h_{AB} = h^{\dagger}_{BA}$. The carbon-carbon dispersion matrices i.e. $\varepsilon_{(A,B)_i(A,B)_j}(\vec{k})$ are

$$h_{AA}(\vec{k}) = \begin{pmatrix} \alpha(\vec{k}) & \beta(\vec{k}) & \gamma(\vec{k}) \\ \beta^*(\vec{k}) & \alpha(\vec{k}) & \theta(\vec{k}) \\ \gamma^*(\vec{k}) & \theta^*(\vec{k}) & \alpha(\vec{k}) \end{pmatrix}, \quad h_{AB}(\vec{k}) = \begin{pmatrix} \tau_1(\vec{k}) & d_2(\vec{k}) & d_3(\vec{k}) \\ d_2(\vec{k}) & \tau_3(\vec{k}) & d_1(\vec{k}) \\ d_3(\vec{k}) & d_1(\vec{k}) & \tau_2(\vec{k}). \end{pmatrix} \tag{A.6}$$

The Li-C dispersion row matrices i.e. $\varepsilon_{LiA_i}(\vec{k})$ and $\varepsilon_{LiB_i}(\vec{k})$ are

$$h_{LiA}(\vec{k}) = [d_{1c}(\vec{k})\ \ d_{3c}(\vec{k})\ \ d_{2c}(\vec{k})] = -t_1^{LiC} e^{ik_z h} \begin{pmatrix} e^{i\vec{k}.\vec{\delta}_1} & e^{i\vec{k}.\vec{\delta}_3} & e^{i\vec{k}.\vec{\delta}_2} \end{pmatrix}, \quad h_{LiB}(\vec{k}) = e^{ik_z h} h_{LiA}{}^*(\vec{k}) \tag{A.7}$$

where $e^{k_z h}$ factor takes 1 by confinement. New variables, $\vec{k}$ dependent on-site energy and chemical potential has been defined as

$$\begin{aligned}
\varepsilon_0(\vec{k}) &= \varepsilon_{Li} - \mu_o + \varepsilon_{LiLi}(\vec{k}) \\
\varepsilon_1(\vec{k}) &= \varepsilon_A - \mu_o + \varepsilon_{A_1A_1}(\vec{k}) \\
\varepsilon_2(\vec{k}) &= \varepsilon_B - \mu_o + \varepsilon_{B_1B_1}(\vec{k})
\end{aligned} \tag{A.8}$$

Shorthand notation has been introduced as follows,

$$\begin{aligned}
\alpha(\vec{k}) \equiv \varepsilon_{A_iA_i}(\vec{k}) &= \varepsilon_{B_iB_i}(\vec{k}) = -t_0 - 2t_5\left(\cos\vec{k}.\vec{\xi}_1 + \cos\vec{k}.\vec{\xi}_2 + \cos\vec{k}.\vec{\xi}_3\right) \\
\beta(\vec{k}) \equiv \varepsilon_{A_1A_2}(\vec{k}) &= \varepsilon^*_{A_2A_1}(\vec{k}) = -t_2 e^{i\vec{k}.(\vec{\delta}_3-\vec{\delta}_1)}\left[1 + w_t\left(e^{-i\vec{k}.\vec{\xi}_3} + e^{i\vec{k}.\vec{\xi}_1}\right)\right] \\
\gamma(\vec{k}) \equiv \varepsilon_{A_1A_3}(\vec{k}) &= \varepsilon^*_{A_3A_1}(\vec{k}) = -t_2 e^{i\vec{k}.(\vec{\delta}_2-\vec{\delta}_1)}\left[1 + w_t\left(e^{-i\vec{k}.\vec{\xi}_2} + e^{i\vec{k}.\vec{\xi}_1}\right)\right] \\
\theta(\vec{k}) \equiv \varepsilon_{A_2A_3}(\vec{k}) &= \varepsilon^*_{A_3A_2}(\vec{k}) = -t_2 e^{i\vec{k}.(\vec{\delta}_2-\vec{\delta}_3)}\left[1 + w_t\left(e^{-i\vec{k}.\vec{\xi}_2} + e^{i\vec{k}.\vec{\xi}_3}\right)\right]
\end{aligned} \tag{A.9}$$

in which $w_t = \frac{t_1^{'}}{t_1} = \frac{t_2^{'}}{t_2}$ and $\vec{\xi}_i = \vec{\tau}_i + 2\vec{\delta}_i$ the d and τ,functions are given by

$$\begin{aligned}
\tau_1(\vec{k}) &= -t_1^{'} e^{i\vec{k}.\vec{\tau}_1}\left[1 + \frac{t_3}{t_1^{'}} e^{-i\vec{k}.\vec{\xi}_1} + \frac{t_4}{t_1^{'}}\left(e^{i\vec{k}.\vec{\xi}_2} + e^{i\vec{k}.\vec{\xi}_3}\right)\right], \quad d_1(\vec{k}) = -t_1 e^{i\vec{k}.\vec{\delta}_1}\left[1 + \frac{t_3}{t_1} e^{-i\vec{k}.\vec{\xi}_1} + \frac{t_4}{t_1}\left(e^{i\vec{k}.\vec{\xi}_2} + e^{i\vec{k}.\vec{\xi}_3}\right)\right] \\
\tau_2(\vec{k}) &= -t_1^{'} e^{i\vec{k}.\vec{\tau}_2}\left[1 + \frac{t_3}{t_1^{'}} e^{-i\vec{k}.\vec{\xi}_2} + \frac{t_4}{t_1^{'}}\left(e^{i\vec{k}.\vec{\xi}_3} + e^{i\vec{k}.\vec{\xi}_1}\right)\right], \quad d_2(\vec{k}) = -t_1 e^{i\vec{k}.\vec{\delta}_2}\left[1 + \frac{t_3}{t_1} e^{-i\vec{k}.\vec{\xi}_2} + \frac{t_4}{t_1}\left(e^{i\vec{k}.\vec{\xi}_3} + e^{i\vec{k}.\vec{\xi}_1}\right)\right] \\
\tau_3(\vec{k}) &= -t_1^{'} e^{i\vec{k}.\vec{\tau}_3}\left[1 + \frac{t_3}{t_1^{'}} e^{-i\vec{k}.\vec{\xi}_3} + \frac{t_4}{t_1^{'}}\left(e^{i\vec{k}.\vec{\xi}_1} + e^{i\vec{k}.\vec{\xi}_2}\right)\right], \quad d_3(\vec{k}) = -t_1 e^{i\vec{k}.\vec{\delta}_3}\left[1 + \frac{t_3}{t_1} e^{-i\vec{k}.\vec{\xi}_3} + \frac{t_4}{t_1}\left(e^{i\vec{k}.\vec{\xi}_1} + e^{i\vec{k}.\vec{\xi}_2}\right)\right].
\end{aligned} \tag{A.10}$$

Also

$$\xi_A(\vec{k}) = \begin{pmatrix} \mathscr{C}_{A_1}(E_i(\vec{k})) \\ \mathscr{C}_{A_2}(E_i(\vec{k})) \\ \mathscr{C}_{A_3}(E_i(\vec{k})) \end{pmatrix} \quad ; \quad \xi_B(\vec{k}) = \begin{pmatrix} \mathscr{C}_{B_1}(E_i(\vec{k})) \\ \mathscr{C}_{B_2}(E_i(\vec{k})) \\ \mathscr{C}_{B_3}(E_i(\vec{k})) \end{pmatrix}. \tag{A.11}$$

Since the Li is an inversion center of the two-dimension tight binding model, the Bloch wave function should respect inversion symmetry. Using $\Psi_{\vec{k}}(\vec{r}) = \langle\vec{r}|\Psi_{\vec{k}}\rangle$, the condition is

$$\Psi_{-\vec{k}}(-\vec{r}) = Ce^{i\phi}\Psi_{\vec{k}}(\vec{r}), \tag{A.12}$$

where C is ± 1 when all subsites in hexagons are carbon, that is $\varepsilon_{A_i} = \varepsilon_{B_i}$. This condition is satisfied if $\mathscr{C}_{A_i}$ is proportional to $\mathscr{C}^*_{B_i}$

$$\mathscr{C}_{A_m}(E_i(\vec{k})) = f_i(\vec{k})\mathscr{C}^*_{B_m}(E_i(\vec{k})), \quad \text{and } \mathscr{C}_{Li}(E_i(\vec{k})) = f_i(\vec{k})\mathscr{C}^*_{Li}(E_i(\vec{k})), \quad i = 1,2,...,7 \tag{A.13}$$

where $f_i(\vec{k})$ is a coefficient to be determined. By inserting $\mathscr{C}_{A_i} = |\mathscr{C}_{A_i}|e^{i\phi_{A_i}}$ and $\mathscr{C}_{B_i} = |\mathscr{C}_{B_i}|e^{i\phi_{B_i}}$ into Eq. A.13 we have

$$f_i(\vec{k}) = \frac{|\mathscr{C}_{A_m}(E_i(\vec{k}))|}{|\mathscr{C}_{B_m}(E_i(\vec{k}))|} e^{i(\phi_{A_i}+\phi_{B_i})} = C_i e^{i\phi_i}, \quad i = 1,2,...,7. \tag{A.14}$$

In the next subsection we use Eqs. A.13 and A.14 to reduce the eigenvalue Eq. A.3 in matrix form to 3×3 to obtain uncoupled shrunken graphene band structure and Bloch wave function coefficients.

B Uncoupled C_6 Dispersion Relations

By first neglecting the lithium-carbon hopping $t_1^{LiC} \to 0$, the shrunken graphene Hamiltonian Eq. A.4 can be diagonalized exactly. Our notation is

$$\left(\begin{array}{c|ccc|ccc} \varepsilon_0(\vec{k}) & 0 & 0 & 0 & 0 & 0 & 0 \\ \hline 0 & \varepsilon_1(\vec{k}) & \beta(\vec{k}) & \gamma(\vec{k}) & \tau_1(\vec{k}) & d_2(\vec{k}) & d_3(\vec{k}) \\ 0 & \beta^*(\vec{k}) & \varepsilon_1(\vec{k}) & \theta(\vec{k}) & d_2(\vec{k}) & \tau_3(\vec{k}) & d_1(\vec{k}) \\ 0 & \gamma^*(\vec{k}) & \theta^*(\vec{k}) & \varepsilon_1(\vec{k}) & d_3(\vec{k}) & d_1(\vec{k}) & \tau_2(\vec{k}) \\ \hline 0 & \tau_1^*(\vec{k}) & d_2^*(\vec{k}) & d_3^*(\vec{k}) & \varepsilon_2(\vec{k}) & \beta^*(\vec{k}) & \gamma^*(\vec{k}) \\ 0 & d_2^*(\vec{k}) & \tau_3^*(\vec{k}) & d_1^*(\vec{k}) & \beta(\vec{k}) & \varepsilon_2(\vec{k}) & \theta^*(\vec{k}) \\ 0 & d_3^*(\vec{k}) & d_1^*(\vec{k}) & \tau_2^*(\vec{k}) & \gamma(\vec{k}) & \theta(\vec{k}) & \varepsilon_2(\vec{k}) \end{array}\right) \left(\begin{array}{c} C_{Li}(E_i^0(\vec{k})) \\ \hline C_{A_1}(E_i^0(\vec{k})) \\ C_{A_2}(E_i^0(\vec{k})) \\ C_{A_3}(E_i^0(\vec{k})) \\ C_{B_1}(E_i^0(\vec{k})) \\ C_{B_2}(E_i^0(\vec{k})) \\ C_{B_3}(E_i^0(\vec{k})) \end{array}\right) = E_i^0(\vec{k}) \left(\begin{array}{c} C_{Li}(E_i^0(\vec{k})) \\ \hline C_{A_1}(E_i^0(\vec{k})) \\ C_{A_2}(E_i^0(\vec{k})) \\ C_{A_3}(E_i^0(\vec{k})) \\ C_{B_1}(E_i^0(\vec{k})) \\ C_{B_2}(E_i^0(\vec{k})) \\ C_{B_3}(E_i^0(\vec{k})) \end{array}\right) \quad \text{(B.1)}$$

The non trivial eigenvalues of uncoupled Hamiltonian Eq. B.1 are given by

$$E_{sh;n}(\vec{k}) = E_{sh,ml}(t_i, \vec{\xi}_i, \vec{k}) = -\mu_o + \alpha(\vec{k}) + u_m \Pi_0(\vec{k}) + u_m^* \Pi_0^*(\vec{k}) + \frac{1}{2}\left[\varepsilon_A + \varepsilon_B + (-1)^l \sqrt{(\varepsilon_A - \varepsilon_B)^2 + 4w_m(\vec{k})}\right] \quad \text{(B.2)}$$

where n is band index defined as

$$n = (2l+1) + (-1)^l m; \qquad m = \begin{cases} 0,\ 1\ ,\ 2 & \text{for l=0} \quad \text{conduction} \\ -1, -2, -3 & \text{for l=1} \quad \text{valence} \end{cases} \quad \text{(B.3)}$$

Here $E_{sh,1}$, $E_{sh,2}$ and $E_{sh,3}$ are conduction bands which corresponds to $l = 0$ and $m = 0,1,2$ and $E_{sh,4}$, $E_{sh,5}$ and $E_{sh,6}$ are valence bands which correspond to $l = 1$, $m = -1,-2,-3$.

At the Γ point, when $\varepsilon_A = \varepsilon_B = \varepsilon^c$, the shrunk graphene eigenstates $|\phi_n(0)\rangle = (C_{A_1}\ C_{A_2}\ C_{A_3}\ C_{B_1}\ C_{B_2}\ C_{B_3})^T$ over the hexagonal subsites take the forms similar to conventional s, d and p orbitals,

$$\begin{aligned} |f\rangle &= (1\ 1\ 1 -1 -1 -1)^T, \quad |p_x\rangle = (1 -1\ 0 -1\ 1\ 0)^T, \quad |p_y\rangle = (1\ 1 -2 -1 -1\ 2)^T, \\ \left|d_{x^2-y^2}\right\rangle &= (1\ 1 -2\ 1\ 1 -2)^T, \quad |d_{xy}\rangle = (1 -1\ 0\ 1 -1\ 0)^T, \quad |S\rangle = (1\ 1\ 1\ 1\ 1\ 1)^T, \end{aligned} \quad \text{(B.4)}$$

with energies,

$$\begin{aligned} E_f &= E_\gamma^+(0) = \mu_s + [(t_1' + 2t_1) + 3t_3 + 6t_4] \\ E_s &= E_\gamma^-(0) = \mu_s - [(t_1' + 2t_1) + 3t_3 + 6t_4] \\ E_p &= E_\alpha^+(0) = E_\beta^+(0) = \mu_d + (t_1' - t_1) \\ E_d &= E_\alpha^-(0) = E_\beta^-(0) = \mu_d - (t_1' - t_1) \end{aligned} \quad \text{(B.5)}$$

where $\mu_s = \varepsilon^c - \mu_0 - 2(t_2 + 2t_2') - 6t_5$ and $\mu_d = \varepsilon^c - \mu_0 + (t_2 + 2t_2') - 6t_5$. For general $\vec{k}$ the uncoupled shrunken graphene eigenfunction Eq. B.24 can be written in terms of $|S\rangle$, $|f\rangle$, $|p_x\rangle$,$|p_y\rangle$, $|d_{xy}\rangle$ and $\left|d_{x^2-y^2}\right\rangle$ as

$$\left|\phi_n(\vec{k})\right\rangle = \left(f_s^n(\vec{k})\,|S\rangle + f_f^n(\vec{k})\,|f\rangle\right) + \left(f_{p_y}^n(\vec{k})\,|p_y\rangle + i f_{d_{xy}}^n(\vec{k})\,|d_{xy}\rangle\right) + \left(f_{d_{x^2-y^2}}^n(\vec{k})\left|d_{x^2-y^2}\right\rangle + i f_{p_x}^n(\vec{k})\,|p_x\rangle\right). \quad \text{(B.6)}$$

In the particular case of pristine graphene in which $w_t = 1$ and $\vec{\tau}_1 = \vec{\delta}_1, \vec{\tau}_2 = \vec{\delta}_2, \vec{\tau}_3 = \vec{\delta}_3$, hence $\beta = \theta = \gamma^*$ and also $\varepsilon_A = \varepsilon_B$ so $C = \pm 1$. Therefore eigenvectors take following form

$$\left|\varphi_{m,l}^0(\vec{k})\right\rangle = \frac{1}{\sqrt{6}}\left(\omega_m\ \ \omega_m{}^*\ \ 1\ \ (-1)^l \frac{\eta_m^*}{|\eta_m|}\omega_m{}^*\ \ (-1)^l \frac{\eta_m^*}{|\eta_m|}\omega_m\ \ (-1)^l \frac{\eta_m^*}{|\eta_m|}\right)^T, \quad \omega_m = e^{i2\pi m/3}, \quad \text{(B.7)}$$

where $m = 1,2,3;\ \ l = 0,1$ and $\eta_m(\vec{k}) = d_2(\vec{k}) + \omega_m d_1(\vec{k}) + \omega_m^* d_3(\vec{k})$. The eigenvalues are

$$E_{m,l}^0 = \varepsilon_{A_1A_1}(\vec{k}) + \omega_m \beta(\vec{k}) + \omega_m^* \beta^*(\vec{k}) + (-1)^l t_1 |\eta_m(\vec{k})| \quad \text{(B.8)}$$

By comparing pristine eigenvectors Eq. B.7 with general shrunken graphene eigenvectors Eq. B.6 it is found that for $m = 1$, $f_{d_{x^2-y^2}}^1(\vec{k}) = -f_{p_x}^1(\vec{k}) = \frac{(1-e^{i\phi_1})}{2\sqrt{2}}$ which corresponds to $d_{x^2-y^2} - ip_x$, also $f_{p_y}^1(\vec{k}) = -f_{d_{xy}}^1(\vec{k}) = \frac{(1+e^{i\phi_1})}{2\sqrt{2}}$ which corresponds to $p_y - id_{xy}$, and the coefficients of s and f are zero. For $m = 2$, $f_{d_{x^2-y^2}}^2(\vec{k}) = f_{p_x}^2(\vec{k}) = \frac{(1-e^{i\phi_2})}{2\sqrt{2}}$ which corresponds to $d_{x^2-y^2} + ip_x$

also $f^2_{p_y}(\vec{k}) = f^2_{d_{xy}}(\vec{k}) = \frac{(1+e^{i\phi_2})}{2\sqrt{2}}$ which corresponds to $p_y + id_{xy}$ while s and f coefficients are zero. For $m = 3$, $f^3_s(\vec{k}) = \frac{(1-e^{i\phi_3})}{2}$ and $f^3_f(\vec{k}) = \frac{(1+e^{i\phi_3})}{2}$ and other coefficients are zero. Here, $e^{i\phi_m} = \frac{\eta^*_m}{|\eta_m|}$.

The Hamiltonian $\hat{H}^{shr}_N$ for the broken symmetry (shrunk graphene) is 6×6 in terms of 3×3 subblocks

$$\begin{pmatrix} h_{AA}(\vec{k}) + \varepsilon_A - \mu_o & h_{AB}(\vec{k}) \\ h_{BA}(\vec{k}) & h^*_{AA}(\vec{k}) + \varepsilon_B - \mu_o \end{pmatrix} \begin{pmatrix} \xi^0_A(E_{sh;i}(\vec{k})) \\ \xi^0_B(E_{sh;i}(\vec{k})) \end{pmatrix} = E_{sh;i}(\vec{k}) \begin{pmatrix} \xi^0_A(E_{sh;i}(\vec{k})) \\ \xi^0_B(E_{sh;i}(\vec{k})) \end{pmatrix} \tag{B.9}$$

To solve Schrödinger Eq. B.9 we first separate the left hand side of Eq. B.9 into two terms $H_{sh}(\vec{k}) = H^0_{sh}(\vec{k}) + H^1_{sh}(\vec{k})$ where

$$H^0_{sh}(\vec{k}) = \begin{pmatrix} \varepsilon_1(\vec{k}) I_{3\times 3} & h_{AB}(\vec{k}) \\ h_{BA}(\vec{k}) & \varepsilon_2(\vec{k}) I_{3\times 3} \end{pmatrix}, \quad H^1_{sh}(\vec{k}) = \begin{pmatrix} h'_{aa}(\vec{k}) & 0_{3\times 3} \\ 0_{3\times 3} & h'_{bb}(\vec{k}) \end{pmatrix} \tag{B.10}$$

where $h'_{aa}(\vec{k}) = (h_{AA}(\vec{k}) - \alpha(\vec{k}) I_{3\times 3})$. In the nearest neighbor approximation it is straightforward to show that $H^0_{sh}(\vec{k})$ and $H^1_{sh}(\vec{k})$ commute with each other, hence they have the same eigenvectors. In more generality one can consider approximately the eigenvectors of H^1_{sh} to be the same as the eigenvectors of H^0_{sh}, therefore one obtains

$$\xi^0_B(\vec{k}) = Ce^{i\phi}\xi^{0*}_A: \quad E_{sh}(\vec{k}) \approx E^0_{sh}(\vec{k}) + E^1_{sh}(\vec{k}). \tag{B.11}$$

with the first equation arising from similar conditions as for Eq. A.13. To find $E_{sh}(\vec{k})$ we solve the following eigenvalue problems

$$\begin{pmatrix} h'_{aa}(\vec{k}) & 0_{3\times 3} \\ 0_{3\times 3} & h'^*_{aa}(\vec{k}) \end{pmatrix} \begin{pmatrix} \xi^0_A(\vec{k}) \\ \xi^0_B(\vec{k}) \end{pmatrix} = E^1_{sh}(\vec{k}) \begin{pmatrix} \xi^0_A(\vec{k}) \\ \xi^0_B(\vec{k}) \end{pmatrix} \tag{B.12}$$

and

$$\begin{pmatrix} \varepsilon_1(\vec{k}) I_{3\times 3} & h_{AB}(\vec{k}) \\ h_{BA}(\vec{k}) & \varepsilon_2(\vec{k}) I_{3\times 3} \end{pmatrix} \begin{pmatrix} \xi^0_A(\vec{k}) \\ \xi^0_B(\vec{k}) \end{pmatrix} = E^0_{sh}(\vec{k}) \begin{pmatrix} \xi^0_A(\vec{k}) \\ \xi^0_B(\vec{k}) \end{pmatrix} \tag{B.13}$$

Eq. B.12 converts to following eigenvalue problem $h'_{aa}(\vec{k})\xi^0_A(\vec{k}) = E^1_{sh}(\vec{k})\xi^0_A(\vec{k})$ and its complex conjugate with $A \leftrightarrow B$, defining

$$\begin{aligned} c_0(t_2, \vec{\xi}_i, \vec{k}) &= \beta(\vec{k})\theta(\vec{k})\gamma^*(\vec{k}) + \gamma(\vec{k})\beta^*(\vec{k})\theta^*(\vec{k}), \ c_1(t_2, \vec{\xi}_i, \vec{k}) = \left|\beta(\vec{k})\right|^2 + \left|\theta(\vec{k})\right|^2 + \left|\gamma(\vec{k})\right|^2 \\ \Pi_0(t_2, \vec{\xi}_i, \vec{k}) &= \left(\frac{c_0(t_2,\vec{\xi}_i,\vec{k})}{2} + i\sqrt{\left(\frac{c_1(t_2,\vec{\xi}_i,\vec{k})}{3}\right)^3 - \left(\frac{c_0(t_2,\vec{\xi}_i,\vec{k})}{2}\right)^2} \right)^{1/3}. \end{aligned} \tag{B.14}$$

eigenvalue equation Eq. B.12 has the three different eigenvalues

$$E^1_{sh,m}(t_2, \vec{\xi}_i, \vec{k}) = u_m \Pi_0(t_2, \vec{\xi}_i, \vec{k}) + u^*_m \Pi^*_0(t_2, \vec{\xi}_i, \vec{k}); \qquad u_m = \sqrt[3]{1} = e^{4im\pi/3}; \ m = 1,2,3 \tag{B.15}$$

note that $E^1_{sh,m}(t_2, \vec{\xi}_i, \vec{k})$ is function of second neighbor hopping $t_{<iA_i, jA_j>} = t_2$ and $\vec{\xi}_i = \vec{\tau}_i + 2\vec{\delta}_i$ i.e. lattice bases vector and it does not depend on $\vec{\tau}_i$ and $\vec{\delta}_i$ separately. Now we calculate $E^0_{sh}(\vec{k})$. Eq. B.13 can be separated into following equations

$$h_{AB}(\vec{k})\xi^0_B(\vec{k}) = (E^0_{sh}(\vec{k}) - \varepsilon_1(\vec{k}))\xi^0_A(\vec{k}), \ \ h_{BA}(\vec{k})\xi^0_A(\vec{k}) = (E^0_{sh}(\vec{k}) - \varepsilon_2(\vec{k}))\xi^0_B(\vec{k}). \tag{B.16}$$

By multiplying first equation of Eq. B.16 by h_{BA} and second by h_{AB} we have

$$\begin{aligned} h_{AB}(\vec{k})h_{BA}(\vec{k})\xi^0_A(\vec{k}) &= (E^0_{sh}(\vec{k}) - \varepsilon_1(\vec{k}))(E^0_{sh}(\vec{k}) - \varepsilon_2(\vec{k}))\xi^0_A(\vec{k}) \\ h_{BA}(\vec{k})h_{AB}(\vec{k})\xi^0_B(\vec{k}) &= (E^0_{sh}(\vec{k}) - \varepsilon_1(\vec{k}))(E^0_{sh}(\vec{k}) - \varepsilon_2(\vec{k}))\xi^0_B(\vec{k}). \end{aligned} \tag{B.17}$$

Eq. B.17 is an eigenvalue problem where second equation is just complex conjugated of first one. By defining new matrix $G(\vec{k}) = h_{AB}(\vec{k})h_{BA}(\vec{k})$ and $w_i(\vec{k}) = [E^0_{sh,i}(\vec{k})]^2 - [\varepsilon_1(\vec{k}) + \varepsilon_2(\vec{k})]E^0_{sh,i}(\vec{k}) + \varepsilon_1(\vec{k})\varepsilon_2(\vec{k})$, Eq. B.17 takes the form

$$G(\vec{k})\xi^0_A(E^0_i(\vec{k})) = w_i(\vec{k})\xi^0_A(E^0_i(\vec{k})) \tag{B.18}$$

Schrödinger Eq. B.18 can be solved to find eigenvalues of Eq. B.13 i.e. $E^0_{sh}(\vec{k})$. Defining

$$\begin{aligned}
C_2(t_i,\vec{\xi}_i,\vec{k}) &= G_{11}+G_{22}+G_{33}\\
C_1(t_i,\vec{\xi}_i,\vec{k}) &= |G_{12}|^2+|G_{13}|^2+|G_{23}|^2-(G_{11}G_{22}+G_{11}G_{33}+G_{22}G_{33})\\
C_0(t_i,\vec{\xi}_i,\vec{k}) &= G_{13}(G_{12}G_{23})^*+G^*_{13}(G_{12}G_{23})-G_{11}|G_{23}|^2-G_{22}|G_{13}|^2-G_{33}|G_{12}|^2+G_{11}G_{22}G_{33}
\end{aligned} \tag{B.19}$$

where $G_{ij}=\sum_{m=1}^{3}\varepsilon_{A_iB_m}(\vec{k})\varepsilon_{B_mA_j}(\vec{k})$. Also introducing

$$\begin{aligned}
\Pi_1(t_i,\vec{\xi}_i,\vec{k}) &= \left(Q(t_i,\vec{\xi}_i,\vec{k})+i\sqrt{P(t_i,\vec{\xi}_i,\vec{k})^3-Q(t_i,\vec{\xi}_i,\vec{k})^2}\right)^{\frac{1}{3}}\\
Q(t_i,\vec{\xi}_i,\vec{k}) &= \frac{C_0(t_i,\vec{\xi}_i,\vec{k})}{2}+\frac{C_1(t_i,\vec{\xi}_i,\vec{k})C_2(t_i,\vec{\xi}_i,\vec{k})}{6}+\frac{C_2^3(t_i,\vec{\xi}_i,\vec{k})}{27}\\
P(t_i,\vec{\xi}_i,\vec{k}) &= \frac{C_1(t_i,\vec{\xi}_i,\vec{k})}{3}+\frac{C_2^2(t_i,\vec{\xi}_i,\vec{k})}{9}.
\end{aligned} \tag{B.20}$$

where $t_i=t_{iA\,jB}$ are first, 3rd and 4th neighbor hopping integrals. Hence eigenvalues of Eq. B.13 can be obtained, they are

$$E^0_{sh;m,l}(t_i,\vec{\xi}_i,\vec{k})=\varepsilon_{A_1A_1}(\vec{k})-\mu_o+\frac{1}{2}\left[\varepsilon_A+\varepsilon_B+(-1)^l\sqrt{(\varepsilon_A-\varepsilon_B)^2+4w_m(t_i,\vec{\xi}_i,\vec{k})}\right] \tag{B.21}$$

where $l=0,\ 1$ and $w_m(t_i,\vec{\xi}_i,\vec{k})$ i.e. solutions of Schrödinger Eq. B.18 are

$$w_m(t_i,\vec{\xi}_i,\vec{k})=\frac{C_2(t_i,\vec{\xi}_i,\vec{k})}{3}+u_m\Pi_1(t_i,\vec{\xi}_i,\vec{k})+u^*_m\Pi^*_1(t_i,\vec{\xi}_i,\vec{k})\quad ;u_m=\sqrt[3]{1}=e^{4im\pi/3}\quad ;\ m=1,2,3. \tag{B.22}$$

Hence from Eqs. B.11, B.15 and B.21 eigenvalues of Schrödinger Eq. B.9 can be obtained,

$$E_{sh;m,l}(t_i,\vec{\xi}_i,\vec{k})=E^1_{sh,m}(t_i,\vec{\xi}_i,\vec{k})+\varepsilon_{A_1A_1}(\vec{k})-\mu_o+\frac{1}{2}\left[\varepsilon_A+\varepsilon_B+(-1)^l\sqrt{(\varepsilon_A-\varepsilon_B)^2+4w_m(t_i,\vec{\xi}_i,\vec{k})}\right] \tag{B.23}$$

The corresponding orthogonal eigenvectors are

$$\left|\phi(E_{sh,n}(\vec{k}))\right\rangle=C_{A_3}(E_{sh;n}(\vec{k}))\left[\begin{pmatrix}\frac{C_{A_1}(E_{sh;n}(\vec{k}))}{C_{A_3}(E_{sh;n}(\vec{k}))} & \frac{C_{A_2}(E_{sh;n}(\vec{k}))}{C_{A_3}(E_{sh;n}(\vec{k}))} & 1\end{pmatrix}\ C\frac{\eta^*(E_{sh;n}(\vec{k}))}{|\eta(E_{sh;n}(\vec{k}))|}\begin{pmatrix}\frac{C^*_{A_1}(E_{sh;n}(\vec{k}))}{C^*_{A_3}(E_{sh;n}(\vec{k}))} & \frac{C^*_{A_2}(E_{sh;n}(\vec{k}))}{C^*_{A_3}(E_{sh;n}(\vec{k}))} & 1\end{pmatrix}\right]^T \tag{B.24}$$

where $C_{A_3}(E_{sh,n}(\vec{k}))$ can be found from orthogonality condition. Also,

$$\begin{aligned}
C_{A_1}(E_{sh,i}(\vec{k})) &= \frac{-\left(G_{22}-w_i(\vec{k})\right)G_{13}+G_{12}G_{23}}{\left(G_{11}-w_i(\vec{k})\right)\left(G_{22}-w_i(\vec{k})\right)-|G_{12}|^2}C_{A_3}(E_{sh,i}(\vec{k}))\\
C_{A_2}(E_{sh,i}(\vec{k})) &= \frac{-\left(G_{11}-w_i(\vec{k})\right)G_{23}+G_{21}G_{13}}{\left(G_{11}-w_i(\vec{k})\right)\left(G_{22}-w_i(\vec{k})\right)-|G_{12}|^2}C_{A_3}(E_{sh,i}(\vec{k})).
\end{aligned} \tag{B.25}$$

To find $\xi_B(E^0_n(\vec{k}))$ it has been used symmetry condition in Eq. B.11

$$C_{B_m}(E_{sh;i}(\vec{k}))=Ce^{i\varphi(E_{sh;i}(\vec{k}))}C^*_{A_m}(E_{sh;i}(\vec{k})). \tag{B.26}$$

Replacing Eq. B.26 into second equation of Eq. B.16 we get

$$\begin{aligned}
e^{i\varphi(E_{sh;i}(\vec{k}))} &= \frac{\eta^*(E_{sh;i}(\vec{k}))}{\left|\eta(E_{sh;i}(\vec{k}))\right|}\frac{C_{A_3}(E_{sh;i}(\vec{k}))}{C^*_{A_3}(E_{sh;i}(\vec{k}))}\quad ;\quad C=\frac{E^0_{sh,i}(\vec{k})-\varepsilon_1(\vec{k})}{E^0_{sh,i}(\vec{k})-\varepsilon_2(\vec{k})}\\
\eta(E_{sh;i}(\vec{k})) &= -t_1\left(d_3\frac{C^*_{A_1}(E_{sh;i}(\vec{k}))}{C^*_{A_3}(E_{sh;i}(\vec{k}))}+d_2\frac{C^*_{A_2}(E_{sh;i}(\vec{k}))}{C^*_{A_3}(E_{sh;i}(\vec{k}))}+\tau_2\right).
\end{aligned} \tag{B.27}$$

It is easy to show that $\left|\eta(E_{sh;i}(\vec{k}))\right|=w_i(\vec{k})$

C Coupled Li-C_6 dispersion relations

By applying the following unitary transformation, $P_{0N}^{\dagger}\hat{H}_N P_{0N}\left(P_{0N}^{\dagger}\left|\Psi_{\vec{k},n}(\vec{r})\right\rangle\right)=E_n(\vec{k})P_{0N}^{\dagger}\left|\Psi_{\vec{k},n}(\vec{r})\right\rangle$, where $\hat{P}_{0N}$ is the operator that diagonalize Eq. B.1, the Schrödinger Eq. A.4 is written in a new matrix representation as

$$\left(\begin{array}{c|cccccc} E_{Li,0}(\vec{k}) & \gamma_1(\vec{k}) & \gamma_2(\vec{k}) & \gamma_3(\vec{k}) & \gamma_4(\vec{k}) & \gamma_5(\vec{k}) & \gamma_6(\vec{k}) \\ \hline \gamma_1^*(\vec{k}) & E_{sh;1}(\vec{k}) & 0 & 0 & 0 & 0 & 0 \\ \gamma_2^*(\vec{k}) & 0 & E_{sh;2}(\vec{k}) & 0 & 0 & 0 & 0 \\ \gamma_3^*(\vec{k}) & 0 & 0 & E_{sh;3}(\vec{k}) & 0 & 0 & 0 \\ \gamma_4^*(\vec{k}) & 0 & 0 & 0 & E_{sh;4}(\vec{k}) & 0 & 0 \\ \gamma_5^*(\vec{k}) & 0 & 0 & 0 & 0 & E_{sh;5}(\vec{k}) & 0 \\ \gamma_6^*(\vec{k}) & 0 & 0 & 0 & 0 & 0 & E_{sh;6}(\vec{k}) \end{array}\right)\begin{pmatrix} A_0(E_i(\vec{k})) \\ A_1(E_i(\vec{k})) \\ A_2(E_i(\vec{k})) \\ A_3(E_i(\vec{k})) \\ A_4(E_i(\vec{k})) \\ A_5(E_i(\vec{k})) \\ A_6(E_i(\vec{k})) \end{pmatrix}=E_i(\vec{k})\begin{pmatrix} A_0(E_i(\vec{k})) \\ A_1(E_i(\vec{k})) \\ A_2(E_i(\vec{k})) \\ A_3(E_i(\vec{k})) \\ A_4(E_i(\vec{k})) \\ A_5(E_i(\vec{k})) \\ A_6(E_i(\vec{k})) \end{pmatrix} \quad \text{(C.1)}$$

where the relation between the column matrix eigenstate of Eq. C.1, $A(E_i(\vec{k}))$, and the eigenstates of Eq. A.4, $\mathscr{C}$ is

$$A=P_{0N}^{\dagger}\mathscr{C}, \text{ and } \mathrm{A}_j(E_i(\vec{k}))=\frac{\gamma_j^*}{E_i(\vec{k})-E_{sh,j}(\vec{k})}A_0(E_i(\vec{k})) \quad \text{(C.2)}$$

in which $A_0(E_i(\vec{k}))$ is determined from the normalization condition, and also

$$\gamma_i(\vec{k})=-t_1^{LiC}\sum_{m=1}^{3}(C_{A_m}(E_{sh,i}(\vec{k}))e^{i\vec{k}.\vec{\zeta}_{A_m}}+C_{B_m}(E_{sh,i}(\vec{k}))e^{i\vec{k}.\vec{\zeta}_{B_m}}) \quad \text{(C.3)}$$

where $\vec{\zeta}_{A_m}$ is a vector that connects Li to the A_m carbon atom and $\vec{\zeta}_{B_m}$ is a vector which connects Li to the B_m carbon atom.

At the Γ point, $\gamma_6(0)=-\sqrt{6}\,t_1^{LiC}$ and $\gamma_i(0)=0$ for $i=1,...,5$. These results show that just the isolated intercalant band, $E_{Li,0}(0)$ and the lowest valance band, $E_{sh,6}(0)$, are mutually affected. The energies of these bands are, with $E_0(0)\equiv E_+$, $E_6(0)\equiv E_-$,

$$E_{\pm}(0)=\frac{1}{2}\left(E_{Li,0}(0)+E_{sh,6}(0)\right)\pm\sqrt{\frac{1}{4}\left[E_{Li,0}(0)-E_{sh,6}(0)\right]^2+6(t_1^{LiC})^2} \quad \text{(C.4)}$$

and other shrunk graphene bands Eq. B.4 remain unchanged. This means the energy gap at Γ, $E_g(0)$, depends only on the nearest neighbor hopping difference rather than on t_1^{LiC}. That is because the overlap between the Li s band and the valance band of uncoupled shrunken graphene (which is linear combination of s and f, $|\phi_6(0)\rangle$) is significant while others are zero.

For general $\vec{k}$ vectors it is challenging to obtain an exact analytical expression for the full Hamiltonian in Eq. A.4 and it would not be transparent anyway. One can use perturbation theory to obtain useful results. For $H_{ND}=H_{0D}+H_{1D}$ we can use non degenerate perturbation theory, obtaining

$$H_{ND}(\vec{k})\,|\psi_N(E_i)\rangle=E_i(\vec{k})\,|\psi_N(E_i)\rangle \quad \text{(C.5)}$$

where expansion of $E_i(\vec{k})$ in terms of perturbation parameter is $E_i(\vec{k})=E_i^0(\vec{k})+\mathscr{E}_i^1(\vec{k})+\mathscr{E}_i^2(\vec{k})+....$ From non degenerate perturbation we have

$$\mathscr{E}_i^1(\vec{k})=\langle\phi_i\,|H_1D|\,\phi_i\rangle\,,\quad \mathscr{E}_i^2(\vec{k})=\sum_{j\neq i}\frac{\left|\langle\phi_i\,|H_1D|\,\phi_j\rangle\right|^2}{E_i^0(\vec{k})-E_j^0(\vec{k})} \quad \text{(C.6)}$$

where $|\phi_i\rangle$ is ith eigenstate of diagonal H_{0D}. Perturbed system eigenstates up to first order are

$$|\psi_N(E_i(\vec{k}))>=\left|\phi_i(E_i^0(\vec{k})\right\rangle+\sum_{j\neq i}\frac{1}{E_i^0-E_j^0}\left|\phi_j^0(E_j(\vec{k})\right\rangle. \quad \text{(C.7)}$$

Non degenerate perturbation theory can be used in Eq. C.1 for completely filled or empty bands that are far from lithium band, $E_{Li,0}(\vec{k})$, and also without overlap. Therefore, except $E_{sh,2}$ and $E_{sh,3}$ which are nearly degenerate with lithium band in some regions, non degenerate approximation can be used for other bands of H_{0N}. We denote Hamiltonian in Eq. C.1 as H_{DN}. This

Hamiltonian can be separated to $H_{ND} = H_{0D} + H_{1D}$ where

$$H_{0D} = \left(\begin{array}{c|cccccc} E_{Li,0}(\vec{k}) & 0 & \gamma_2(\vec{k}) & \gamma_3(\vec{k}) & 0 & 0 & 0 \\ \hline 0 & E_{sh,1}(\vec{k}) & 0 & 0 & 0 & 0 & 0 \\ \gamma_2^*(\vec{k}) & 0 & E_{sh,2}(\vec{k}) & 0 & 0 & 0 & 0 \\ \gamma_3^*(\vec{k}) & 0 & 0 & E_{sh,3}(\vec{k}) & 0 & 0 & 0 \\ 0 & 0 & 0 & 0 & E_{sh,4}(\vec{k}) & 0 & 0 \\ 0 & 0 & 0 & 0 & 0 & E_{sh,5}(\vec{k}) & 0 \\ 0 & 0 & 0 & 0 & 0 & 0 & E_{sh,6}(\vec{k}) \end{array}\right). \tag{C.8}$$

$$H_{1D} = \left(\begin{array}{c|cccccc} 0 & \gamma_1(\vec{k}) & 0 & 0 & \gamma_4(\vec{k}) & \gamma_5(k) & \gamma_6(\vec{k}) \\ \hline \gamma_1^*(\vec{k}) & 0 & 0 & 0 & 0 & 0 & 0 \\ 0 & 0 & 0 & 0 & 0 & 0 & 0 \\ 0 & 0 & 0 & 0 & 0 & 0 & 0 \\ \gamma_4^*(\vec{k}) & 0 & 0 & 0 & 0 & 0 & 0 \\ \gamma_5^*(\vec{k}) & 0 & 0 & 0 & 0 & 0 & 0 \\ \gamma_6^*(\vec{k}) & 0 & 0 & 0 & 0 & 0 & 0 \end{array}\right). \tag{C.9}$$

introducing below coefficients

$$\begin{aligned} c_2 &= E_{Li,0}(\vec{k}) + E_{sh,2}(\vec{k}) + E_{sh,3}(\vec{k}) \\ c_1 &= -(E_{Li,0}(\vec{k})E_{sh,2}(\vec{k}) + E_{Li,0}(\vec{k})E_{sh,3}(\vec{k}) + E_{sh,2}(\vec{k})E_{sh,3}(\vec{k}) - |\gamma_2(\vec{k})|^2 - |\gamma_3(\vec{k})|^2) \\ c_0 &= E_{Li,0}(\vec{k})E_{sh,2}(\vec{k})E_{sh,3}(\vec{k}) - E_{sh,3}(\vec{k})|\gamma_2(\vec{k})|^2 - E_{sh,2}(\vec{k})|\gamma_3(\vec{k})|^2 \\ \Pi &= (q + i\sqrt{p^3 - q^2}),\ \ q = \frac{c_0}{2} + \frac{c_1 c_2}{6} + \frac{c_2^3}{27},\ \ \ p = \frac{c_1}{3} + \frac{c_2^2}{9} \end{aligned} \tag{C.10}$$

Non trivial eigenstate of H_{0D} are

$$E_0^0(\vec{k}) = \frac{c_2}{2} + \Pi + \Pi^*,\ \ E_2^0(\vec{k}) = \frac{c_2}{2} + e^{i2\pi/3}\Pi + e^{-i2\pi/3}\Pi^*,\ \ E_3^0(\vec{k}) = \frac{c_2}{2} + e^{-i2\pi/3}\Pi + e^{i2\pi/3}\Pi^* \tag{C.11}$$

and corresponding eigenstates are

$$\begin{aligned} \left|\phi_0(E_0^0(\vec{k}))\right\rangle &= (C_{p1}(E_0^0(\vec{k}))\ 0\ C_{p2}(E_0^0(\vec{k}))C_{p3}(E_0^0(\vec{k}))0\ \ 0\ \ 0)^T \\ \left|\phi_2(E_2^0(\vec{k}))\right\rangle &= (C_{p1}(E_2^0(\vec{k}))\ 0\ C_{p2}(E_2^0(\vec{k}))C_{p3}(E_2^0(\vec{k}))0\ \ 0\ \ 0)^T \\ \left|\phi_3(E_3^0(\vec{k}))\right\rangle &= (C_{p1}(E_3^0(\vec{k}))\ 0\ C_{p2}(E_3^0(\vec{k}))C_{p3}(E_3^0(\vec{k}))0\ \ 0\ \ 0)^T \end{aligned} \tag{C.12}$$

It is easy to show that $\mathscr{E}_1^0 = 0$. So up to second order perturbation parameter the eigenenergies are

$$\begin{aligned}
E_0(\vec{k}) &= E_0^0(\vec{k}) - |C_{p1}(E_0^0)|^2 \left(\frac{|\gamma_1|^2}{E_{sh,1}-E_0^0} + \frac{|\gamma_4|^2}{E_{sh,4}-E_0^0} + \frac{|\gamma_5|^2}{E_{sh,5}-E_0^0} + \frac{|\gamma_6|^2}{E_{sh,6}-E_0^0} \right) \\
E_1(\vec{k}) &= E_{sh,1}(\vec{k}) + |C_{p1}(E_0^0)|^2 \frac{|\gamma_1|^2}{E_{sh,1}-E_0^0} + |C_{p1}(E_2^0)|^2 \frac{|\gamma_1|^2}{E_{sh,1}-E_2^0} + |C_{p1}(E_3^0)|^2 \frac{|\gamma_1|^2}{E_{sh,1}-E_3^0} \\
E_2(\vec{k}) &= E_2^0(\vec{k}) - |C_{p1}(E_2^0)|^2 \left(\frac{|\gamma_1|^2}{E_{sh,1}-E_2^0} + \frac{|\gamma_4|^2}{E_{sh,4}-E_2^0} + \frac{|\gamma_5|^2}{E_{sh,5}-E_2^0} + \frac{|\gamma_6|^2}{E_{sh,6}-E_2^0} \right) \\
E_3(\vec{k}) &= E_3^0(\vec{k}) - |C_{p1}(E_3^0)|^2 \left(\frac{|\gamma_1|^2}{E_{sh,1}-E_3^0} + \frac{|\gamma_4|^2}{E_{sh,4}-E_3^0} + \frac{|\gamma_5|^2}{E_{sh,5}-E_3^0} + \frac{|\gamma_6|^2}{E_{sh,6}-E_3^0} \right) \\
E_4(\vec{k}) &= E_{sh,4}(\vec{k}) + |C_{p1}(E_0^0)|^2 \frac{|\gamma_4|^2}{E_{sh,4}-E_0^0} + |C_{p1}(E_2^0)|^2 \frac{|\gamma_4|^2}{E_{sh,4}-E_2^0} + |C_{p1}(E_3^0)|^2 \frac{|\gamma_4|^2}{E_{sh,4}-E_3^0} \\
E_5(\vec{k}) &= E_{sh,5}(\vec{k}) + |C_{p1}(E_0^0)|^2 \frac{|\gamma_5|^2}{E_{sh,5}-E_0^0} + |C_{p1}(E_2^0)|^2 \frac{|\gamma_5|^2}{E_{sh,5}-E_2^0} + |C_{p1}(E_3^0)|^2 \frac{|\gamma_5|^2}{E_{sh,5}-E_3^0} \\
E_6(\vec{k}) &= E_{sh,6}(\vec{k}) + |C_{p1}(E_0^0)|^2 \frac{|\gamma_6|^2}{E_{sh,6}-E_0^0} + |C_{p1}(E_2^0)|^2 \frac{|\gamma_6|^2}{E_{sh,6}-E_2^0} + |C_{p1}(E_3^0)|^2 \frac{|\gamma_6|^2}{E_{sh,6}-E_3^0}
\end{aligned} \tag{C.13}$$

D Bogoliubov-de Gennes Transformation

The electron-electron interaction part of Hamiltonian, H_P, in the mean field approximation becomes

$$\hat{H}_P^{MF} = \frac{1}{2} \sum_{i\alpha\sigma} \sum_{j\beta\sigma'} \Delta_{i\alpha j\beta}^{\sigma\sigma'} \hat{c}_{i\alpha\sigma}^{\dagger} \hat{c}_{j\beta\sigma'}^{\dagger} + h.c. + F_0 = \frac{1}{2} \sum_{\vec{k}\alpha\sigma\beta\sigma'} \Delta_{\alpha\beta}^{\sigma\sigma'}(\vec{k}) \hat{c}_{\alpha\sigma}^{\dagger}(\vec{k}) \hat{c}_{\beta\sigma'}^{\dagger}(-\vec{k}) + h.c. + F_0 \tag{D.1}$$

in which $\Delta_{i\alpha j\beta}^{\sigma\sigma'} = U_{i\alpha,j\beta}^{\sigma\sigma'} \left\langle \hat{c}_{i\alpha\sigma} \hat{c}_{j\beta\sigma'} \right\rangle$ is the matrix of order parameters in real space. Fourier transformation of real space order parameters are given by

$$\Delta_{\alpha\beta}^{\sigma\sigma'}(\vec{k}) = \frac{1}{N} \sum_{ij} \Delta_{i\alpha j\beta}^{\sigma\sigma'} e^{i\vec{k}.(\vec{r}_{i\alpha} - \vec{r}_{j\beta})}. \tag{D.2}$$

here Latin subscripts, α and β refers to A_i or B_i subsites. The interacting Hamiltonian in Nambu space is

$$\hat{H}_{SU} = \sum_{\vec{k}} \hat{\Psi}^{\dagger}(\vec{k}) H_{SU}(\vec{k}) \hat{\Psi}(\vec{k}) \tag{D.3}$$

where $\hat{\Psi}^{\dagger}(\vec{k}) = (c_{0\uparrow}^{\dagger}(\vec{k})\ c_{1\uparrow}^{\dagger}(\vec{k})\ ,...,\ c_{6\uparrow}^{\dagger}(\vec{k})\ c_{0\downarrow}(-\vec{k})\ c_{1\downarrow}(-\vec{k})\ ,...,c_{6\downarrow}(-\vec{k}))$ where Li, A_1, A_2, A_3, B_1, B_2 and B_3 are labeled by $0,\ 1,2,3,\ 4,5,6$ respectively, and

$$H_{SU}(\vec{k}) = \begin{pmatrix} H_N(\vec{k}) & H_P(\vec{k}) \\ H_P^{\dagger}(\vec{k}) & -H_N^*(-\vec{k}) \end{pmatrix}. \tag{D.4}$$

The coupling is given by

$$H_P(\vec{k}) = \left(\begin{array}{c|ccc|ccc}
0 & 0 & 0 & 0 & 0 & 0 & 0 \\
\hline
0 & 0 & 0 & 0 & \Delta_{A_1B_1}^{\uparrow\downarrow}(\vec{k}) & \Delta_{A_1B_2}^{\uparrow\downarrow}(\vec{k}) & \Delta_{A_1B_3}^{\uparrow\downarrow}(\vec{k}) \\
0 & 0 & 0 & 0 & \Delta_{A_2B_1}^{\uparrow\downarrow}(\vec{k}) & \Delta_{A_2B_2}^{\uparrow\downarrow}(\vec{k}) & \Delta_{A_2B_3}^{\uparrow\downarrow}(\vec{k}) \\
0 & 0 & 0 & 0 & \Delta_{A_3B_1}^{\uparrow\downarrow}(\vec{k}) & \Delta_{A_3B_2}^{\uparrow\downarrow}(\vec{k}) & \Delta_{A_3B_3}^{\uparrow\downarrow}(\vec{k}) \\
\hline
0 & \Delta_{A_1B_1}^{\uparrow\downarrow *}(\vec{k}) & \Delta_{A_2B_1}^{\uparrow\downarrow *}(\vec{k}) & \Delta_{A_3B_1}^{\uparrow\downarrow *}(\vec{k}) & 0 & 0 & 0 \\
0 & \Delta_{A_1B_2}^{\uparrow\downarrow *}(\vec{k}) & \Delta_{A_2B_2}^{\uparrow\downarrow *}(\vec{k}) & \Delta_{A_3B_2}^{\uparrow\downarrow *}(\vec{k}) & 0 & 0 & 0 \\
0 & \Delta_{A_1B_3}^{\uparrow\downarrow *}(\vec{k}) & \Delta_{A_2B_3}^{\uparrow\downarrow *}(\vec{k}) & \Delta_{A_3B_3}^{\uparrow\downarrow *}(\vec{k}) & 0 & 0 & 0
\end{array}\right) \tag{D.5}$$

for singlet $\Delta^{\uparrow\downarrow}_{\beta\alpha}(\vec{k}) = \Delta^{\uparrow\downarrow}_{\alpha\beta}(-\vec{k}) = -\Delta^{\downarrow\uparrow}_{\alpha\beta}(-\vec{k}) = \Delta^{\uparrow\downarrow\,*}_{\alpha\beta}(\vec{k})$. The order parameters according to (main text) Fig. 4 are

$$\begin{aligned}
\Delta^{\uparrow\downarrow}_{A_1B_1}(\vec{k}) &= \Delta''_1 e^{i\vec{k}.\vec{\tau}_1}, \quad \Delta^{\uparrow\downarrow}_{A_1B_2}(\vec{k}) = \Delta_2 e^{i\vec{k}.\vec{\delta}_2}, \quad \Delta^{\uparrow\downarrow}_{A_1B_3}(\vec{k}) = \Delta'_3 e^{i\vec{k}.\vec{\delta}_3};\\
\Delta^{\uparrow\downarrow}_{A_2B_1}(\vec{k}) &= \Delta'_2 e^{i\vec{k}.\vec{\delta}_2}, \quad \Delta^{\uparrow\downarrow}_{A_2B_2}(\vec{k}) = \Delta''_3 e^{i\vec{k}.\vec{\tau}_3}, \quad \Delta^{\uparrow\downarrow}_{A_2B_3}(\vec{k}) = \Delta_1 e^{i\vec{k}.\vec{\delta}_1};\\
\Delta^{\uparrow\downarrow}_{A_3B_1}(\vec{k}) &= \Delta_3 e^{i\vec{k}.\vec{\delta}_3}, \quad \Delta^{\uparrow\downarrow}_{A_3B_2}(\vec{k}) = \Delta'_1 e^{i\vec{k}.\vec{\delta}_1}, \quad \Delta^{\uparrow\downarrow}_{A_3B_3}(\vec{k}) = \Delta''_2 e^{i\vec{k}.\vec{\tau}_2}.
\end{aligned} \tag{D.6}$$

$U_{<iA_1\uparrow jB_1\downarrow>} = U_{<iA_2\uparrow jB_2\downarrow>} = U_{<iA_3\uparrow jB_3\downarrow>} g_1$ Quasiparticle energies are obtained by unitary transformation in the seven band space

$$\hat{H}_{SU} = \sum_{\vec{k}} \hat{\Psi}^\dagger(\vec{k}) Q \left[Q^\dagger H_{SU}(\vec{k}) Q \right] Q^\dagger \hat{\Psi}(\vec{k}) = \sum_{\vec{k}} \Lambda^\dagger(\vec{k}) H^N_{SU}(\vec{k}) \Lambda(\vec{k}) \tag{D.7}$$

where in matrix notation,

$$H^N_{SU}(\vec{k}) = \begin{pmatrix} H_{ND}(k) & H_{PD}(\vec{k}) \\ H^\dagger_{PD}(\vec{k}) & -H^*_{ND}(-\vec{k}) \end{pmatrix}, \quad Q = \begin{pmatrix} \hat{P}_N(\vec{k}) & \widehat{0} \\ \widehat{0} & \hat{P}^*_N(-\vec{k}) \end{pmatrix}. \tag{D.8}$$

$\hat{P}_N(\vec{k})$ is a 7×7 matrix where each column is one of the perturbed normal state eigenvectors of Eq. A.4, thus with matrix elements given by $[\hat{P}^\dagger_N]_{i,j} = \mathscr{C}_i(E_j(\vec{k}))$. $H_{ND}(\vec{k})$ is the corresponding diagonal seven band Hamiltonian. Here $\hat{P}^*_N(-\vec{k}) = \hat{P}_N(\vec{k})$. In the normal band space the matrix elements of the off-diagonal array are given by

$$[H_{DP}(\vec{k})]_{i,j} = \Delta_{ij}(\vec{k}) = \sum_{\alpha=1}^{9} \Omega^\alpha_{ij}(\vec{k}) \Delta^\alpha \tag{D.9}$$

Using the fact that the gap is small, applying perturbation up to second order in the order parameter gives quasiparticle energies

$$E^Q_{m,s}(\vec{k}) = s\left(E_m(\vec{k}) + \sum_{i=1}^{7} \frac{\left|\Delta_{mi}(\vec{k})\right|^2}{E_m(\vec{k}) + E_i(\vec{k})} \right) \qquad s = \pm 1 \tag{D.10}$$

where $s = 1$ is for particles and $s = -1$ for holes.

E Superconducting States

By minimizing the quasiparticle free energy with respect to nearest neighbor order parameters the gap equation is obtained. The free energy is

$$F = -\frac{2}{\beta} \sum_{\vec{k}} \sum_{n=1}^{7} \ln\left[2\cosh(\frac{E^Q_n}{2k_BT}) \right] + F_0. \tag{E.1}$$

where F_0 is the system condensation energy,

$$F_0 = -\frac{1}{2} \sum_{i\alpha\sigma} \sum_{j\beta\sigma'} \Delta^{\sigma\sigma'}_{i\alpha j\beta} G^{\sigma\sigma'\,\dagger}_{i\alpha j\beta} = -2N \sum_{\alpha=1}^{9} J_\alpha (\Delta^\alpha)^2 \tag{E.2}$$

where $J_1 = J_2 = J_3 = \frac{1}{g_1}$ and $J_4 = J_5 = J_6 = J_7 = J_8 = J_9 = \frac{1}{g_0}$. The linearized gap equation, obtained by minimizing free energy of the system, is

$$J_\beta \Delta^\beta = -\frac{1}{2N} \sum_{\alpha=1}^{9} \left[\sum_{\vec{k}} \sum_{n=1}^{7} \sum_{i=1}^{7} \frac{\tanh(\frac{E^Q_n}{2k_BT})}{E_n(\vec{k}) + E_i(\vec{k})} \left(\Omega^\alpha_{ni}(\vec{k}) \Omega^{*\beta}_{ni}(\vec{k}) + \Omega^\beta_{ni}(\vec{k}) \Omega^{*\alpha}_{ni}(\vec{k}) \right) \right] \Delta^\alpha \equiv -\sum_{\alpha=1}^{9} \Gamma_{\beta\alpha} \Delta^\alpha. \tag{E.3}$$

We have used that at T_c, where $|\Delta_{ij}|^2$ can be neglected, $E^Q_n \to E_n$.

In the general case, (see main text Fig. 4) , the system is invariant under interchange $2 \rightleftharpoons 5$, $3 \rightleftharpoons 6$ and $4 \rightleftharpoons 7$, which means $\Delta'_i \rightleftharpoons \Delta_i$. These relations correspond to $\Delta^4 \rightleftharpoons \Delta^7$, $\Delta^5 \rightleftharpoons \Delta^8$ and $\Delta^6 \rightleftharpoons \Delta^9$ in Eq. E.3. This symmetry and that of the symmetric Γ matrix $\Gamma_{\beta\alpha} = \Gamma_{\alpha\beta}$ allows Eq. E.3 to be written in matrix form as

$$\begin{bmatrix} A_{3\times3} & B_{3\times3} & B_{3\times3} \\ B_{3\times3} & C_{3\times3} & D_{3\times3} \\ B_{3\times3} & D_{3\times3} & C_{3\times3} \end{bmatrix} \begin{pmatrix} g_1 V_1 \\ g_0 V_2 \\ g_0 V_3 \end{pmatrix} = -\begin{pmatrix} V_1 \\ V_2 \\ V_3 \end{pmatrix} \tag{E.4}$$

where

$$A_{3\times3} = \begin{bmatrix} \Gamma_{11} & \Gamma_{12} & \Gamma_{12} \\ \Gamma_{12} & \Gamma_{11} & \Gamma_{12} \\ \Gamma_{12} & \Gamma_{12} & \Gamma_{11} \end{bmatrix}, C_{3\times3} = \begin{bmatrix} \Gamma_{44} & \Gamma_{45} & \Gamma_{45} \\ \Gamma_{45} & \Gamma_{44} & \Gamma_{45} \\ \Gamma_{45} & \Gamma_{45} & \Gamma_{44} \end{bmatrix}, B_{3\times3} = \begin{bmatrix} \Gamma_{14} & \Gamma_{15} & \Gamma_{15} \\ \Gamma_{15} & \Gamma_{14} & \Gamma_{15} \\ \Gamma_{15} & \Gamma_{15} & \Gamma_{14} \end{bmatrix}, D_{3\times3} = \begin{bmatrix} \Gamma_{47} & \Gamma_{48} & \Gamma_{48} \\ \Gamma_{48} & \Gamma_{47} & \Gamma_{48} \\ \Gamma_{48} & \Gamma_{48} & \Gamma_{47} \end{bmatrix} \quad \text{(E.5)}$$

Eq. E.4 can be written as a non-Hermitian eigenvalue problem

$$\begin{bmatrix} \kappa A & \kappa B & \kappa B \\ B & C & D \\ B & D & C \end{bmatrix} \begin{pmatrix} g_1 V_1 \\ g_0 V_2 \\ g_0 V_3 \end{pmatrix} = -\frac{1}{g_0} \begin{pmatrix} g_1 V_1 \\ g_0 V_2 \\ g_0 V_3 \end{pmatrix} \quad \text{(E.6)}$$

where $\kappa = \frac{g_1}{g_0}$. Substates V_1, V_2 and V_3 in gap equation Eq. E.6, cannot be have different symmetries, so each of the eigenvectors of Eq. E.6 can be expressed in the compact form

$$[\Phi_n]^T = [\alpha_{sy} V_{sy} \quad \beta_{sy} V_{sy} \quad \gamma_{sy} V_{sy}] \quad \text{(E.7)}$$

where subscript sy refers to one of the s-wave, d_{xy}-wave or $d_{x^2-y^2}$-wave symmetries, and the coefficients α_{sy}, β_{sy} and γ_{sy} are to be determined. By inserting eigenvectors from Eq. E.7 into the gap equation Eq. E.6 one finds

$$\frac{\alpha_{sy}}{\beta_{sy}} = \frac{\alpha_{sy}}{\gamma_{sy}}, \qquad \frac{\gamma_{sy}}{\beta_{sy}} = \frac{\beta_{sy}}{\gamma_{sy}}, \qquad J_{sy} = c_{sy} + \frac{\beta_{sy}}{\gamma_{sy}} d_{sy} + \frac{\alpha_{sy}}{\gamma_{sy}} b_{sy} \quad \text{(E.8)}$$

where $c_{sy} = c_s, c_d$, $b_{sy} = b_s, b_d$, $d_{sy} = d_s, d_d$ and $J_{sy} = -\frac{1}{g_0}$ for each symmetry. Eq. E.8 has two classes of solutions

$$\begin{aligned} \beta_{sy} &= -\gamma_{sy} \equiv 1 \quad \Rightarrow \quad \alpha_{sy} = 0,\ J^0_{sy} = c_{sy} - d_{sy}, \\ \beta_{sy} &= +\gamma_{sy} \equiv 1 \quad \Rightarrow \quad b_{sy}\alpha^2_{sy} + (c_{sy} + d_{sy} - \kappa a_{sy})\alpha_{sy} - 2\kappa b_{sy} = 0 \end{aligned} \quad \text{(E.9)}$$

In the limiting case of pristine graphene, the quadratic equation in Eq. E.9, has two temperature independent solutions $\alpha_{sy} = 1$ and $\alpha_{sy} = -2$. The last solution in addition to island states $\alpha_{sy} = 0$ are in fact, orthogonal states where they are linear combination of the aforementioned Φ_{0n} and Φ_{1n}. But in the general case of symmetry breaking LiC_6 characterized by gap equation Eq. E.4, the quadratic equation in Eq. E.9, has two temperature dependent solutions,

$$\alpha^{\pm}_{sy} = \frac{J^{\pm}_{sy} - c_{sy} - d_{sy}}{b_{sy}}, \quad J^{\pm}_{sy} = \frac{1}{2}\left(\kappa a_{sy} + c_{sy} + d_{sy} \pm \sqrt{8\kappa b^2_{sy} + [c_{sy} + d_{sy} - \kappa a_{sy}]^2}\right) \quad \text{(E.10)}$$
